# Detecting Adverse Drug Events in Social Media: A Brief Literature Review

**DOI:** 10.1007/s42979-026-04752-9

**Published:** 2026-02-11

**Authors:** Imane Guellil, Yousra Berrachedi, Nidhal Eddine Chenni, Massi-Nissa Abboud, Jinge Wu, Honghan Wu, Beatrice Alex

**Affiliations:** 1 Edinburgh University, Edinburgh, UK; 2 Higher School of Computer Science, Algiers, Algeria; 3 University Cote d’Azur, Cote d’Azur, France; 4 University College London UCL, London, UK; 5 University of Glasgow, Glasgow, UK; 6 University of Edinburgh, Edinburgh, UK

**Keywords:** Adverse drug events, ADEs, Natural language processing, NLP, Social media

## Abstract

Adverse drug events (ADEs) remain a significant burden to public health and a persistent challenge for pharmacovigilance. The proliferation of patient-generated discourse on social media offers a complementary, real-time signal for ADE surveillance. This article provides a concise yet comprehensive review of recent natural language processing (NLP) research on identifying ADEs in social media text. We systematically reviewed 100 peer-reviewed studies (2017–2025) on NLP/AI for detecting or analysing ADEs in social media. Searches in Google Scholar targeted English-language journal and conference papers; patents and protocols were excluded. Of 130 records screened, 6 were protocols and 24 were excluded because the full text could not be located or the item was a conference abstract lacking methodological detail (i.e., no description of approaches or experiments), yielding a final sample of 100 studies. One reviewer performed screening, with full-text eligibility verified by a second. We extracted objectives, data sources/languages, preprocessing and annotation practices, datasets, model families, evaluation metrics, and stated limitations. Studies were grouped into five task categories–classification, extraction, normalization, corpus creation, and broader analytical work–with evidence tables summarizing contributions, toolchains, datasets, and performance. Recurrent challenges include noisy/imbalanced data, multilingual and code-mixed content, and variability in annotation standards. Twitter remains the primary data source: 60% of studies analyse Twitter alone and a further 18% combine Twitter with other platforms (78% in total). English overwhelmingly dominates; only about 5% of studies draw on non-English sources (e.g., French, Chinese, Arabic). Standard pre-processing–URL removal, tokenisation, and lowercasing–is near-universal. Transformer-based models predominate, with BERT and its biomedical or “tweet” variants (e.g., RoBERTa, BioBERT, BERTweet) used in more than 60% of approaches. Persistent obstacles include severe class imbalance and ambiguous or implicit drug-event expressions. Although shared tasks such as SMM4H provide widely used benchmarks, comprehensive annotation guidelines remain uncommon (12% of papers). Recent work increasingly incorporates multimodal inputs and integrates structured biomedical knowledge, yet gaps persist in multilingual coverage, temporal/longitudinal modelling, and real-world deployment. To our knowledge, this is the first review to synthesise findings from a corpus of 100 peer-reviewed studies on ADE detection in social media using NLP. By organising the literature by task type and tracing methodological trends and limitations, it provides practical guidance for researchers and practitioners. The review also outlines actionable directions for future work, including model explainability, support for low-resource languages, and closer collaboration with regulatory authorities to enable real-world deployment.

## Abstract (unstructured)

In this paper, we look at how natural language processing (NLP)–computer methods that read and learn from text–can spot adverse drug events (ADEs) in social media. We reviewed 100 research papers and summarised their aims, approaches, models, datasets, novelty, and limitations. We grouped the work into five practical buckets: classification (deciding if a post mentions an ADE), extraction (pulling out the drug and the event), normalisation (mapping everyday terms to medical ones), corpus creation (building datasets), and broader analysis. We compile the latest techniques and resources in a synthesis table and highlight consistent patterns: Twitter is the most studied platform; simple pre-processing such as removing or replacing URLs is common; and transformer models–especially BERT and its biomedical variants–are widely used. We also flag recurring hurdles: imbalanced data, the difficulty of annotating posts (especially when drug-event links are implied rather than explicit), few papers with clear annotation guidelines, heavy reliance on shared-task datasets like Social Media Mining for Health (SMM4H), and a strong bias toward English with limited work in languages such as French or Chinese. By bringing these findings together–summarising, comparing, and contrasting–we fill a gap in the field and offer a clear, lay-friendly map of current methods, datasets, and benchmarks.

## Introduction

Social media platforms have become integral venues for health-related discourse, providing large-scale patient-generated data on symptoms, medication use, and adverse drug events (ADEs). An adverse drug event is defined as follows:

An Adverse Drug Event (ADE), also known as ADR for Adverse Drug Reaction or drug side-effect, refers to any injuries resulting from medication use, including physical harm, mental harm, or loss of function, that is threatening public health and have become a leading cause of death [72, 81].

The large volume of data generated from social media, along with its relevance, makes it a valuable data source for pharmacological studies. Pharmaceutical companies are increasingly analysing social media posts that describe patient-reported experiences with their products. Due to the scale of data, such knowledge distillation would require the application of Natural Language Processing (NLP) techniques to collect, extract, represent, analyse, and verify data from social media such as Twitter, Reddit, Instagram, Facebook, forums, etc. [24, 33, 55, 67].

The detection of ADEs is a crucial task in the pharmaceutical industry, as ADEs can have a profound impact on patient quality of life and contribute to increased mortality worldwide. With the extensive use of social media and the abundance of health-related discussions, drugs and ADE are some of the most frequently discussed topics. Hence, social media provide excellent data for ADE extractions [26, 37, 64, 96, 102].

Detecting adverse events from social media faces many challenges, including typos, grammar errors, elongation, repeated punctuation, and the use of slang, sarcasm, and irony. The following list presents some examples extracted from social media referring to ADEs that were previously included in [31]:

- One of the things i hate most about quetiapine is when i take it for the first few hours i **slur** my words, so people assume i’m merely drunk.
- Ciprofloxacin: how do you expect to **sleep** when your **stomach is a cement mixer ?**
- Just woke up. since i started on the higher dose of quetiapine i’m **sleeping** even more ...; i feel **knackered when i wake**.

These phenomena also motivate robustness and domain-shift research, cross-platform analyses, and exploration of multilingual or multimodal signals [4, 67, 85, 108].

Different methods have been proposed for classifying, detecting, normalising and analysing ADEs using NLP. For example, studies focusing on classification only detect if a post/comment includes an ADE or not. All of the above examples include ADEs, so the task of a classification system is to detect them as including ADEs. The task of ADE detection consists of extracting different ADEs from the posts/comments. In the previous examples: ‘*slur*’ would be extracted from the first example, ‘*sleep*’ and ‘*stomach is a cement mixer ?*’ from the second example and ‘*sleeping*’ and ‘*knackered when i wake*’ from the third example. The task of normalisation means to map the different extracted ADEs to an existing ontology such as *Unified Medical Language System (UMLS)*,^1^*SNOMED CT*^2^ or to the *Medical dictionary for the Regulatory Activities (MedDRA)*.^3^ For example, if ADEs are mapped to MedDRA, ‘*slur*’ should be associated with Slurred speech, ‘*sleep*’ with Sleeplessness, ‘*stomach is a cement mixer ?*’ with Stomach perforation, ‘*sleeping*’ with Sleepiness and ‘*knackered when i wake*’ with Groggy on awakening. *Recent work has improved normalisation in noisy, informal contexts using sentence-transformer biomedical representations and zero-shot linking, and by coupling extraction and linking in end-to-end pipelines* [104, 140].

Some of the previous work reviewed in this area relies on two or three of these tasks combined in a pipeline that first classifies comments before extracting different ADEs. In some cases, ADEs are also mapped to an ontology after being extracted. To classify, detect and normalise ADEs, the majority of the existing research studies rely on machine learning algorithms, which require training data. Hence some of the works mainly focus on the construction of the resources that would be required for training and validating the proposed models. Finally, the last group of reviewed works is dedicated to different analyses related to ADEs and mentioned drugs. These studies also highlight the sentiment and anxiety related to ADEs. *More recently, transformer and LLM-based pipelines, ensembles, and quantum-inspired models have been explored for end-to-end pharmacovigilance on social media and patient reviews* [29, 64, 83, 96, 130, 140].

To summarise, synthesise and classify the different works related to the use of NLP for classifying, detecting, normalising and analysing ADEs. This paper is organised as follows: Sect. 3 is dedicated to the background related to ADEs in social media. Section 4 illustrates and groups the papers reviewed into different categories. Section 5 presents an analysis of the studies works. Section 6 highlights Practical applications of findings in pharmacovigilance and regulatory practices. Section 7 contributes a discussion and presents future direction. of the reviewed research. Finally, Sect. 8 provides a general conclusion by highlighting some insights learned as a result of this review.

## Adverse Drug Events in Social Media: Background

In the United States, ADEs affect hundreds of thousands of people and cost billions of dollars in outpatient settings in the U.S. alone, with these costs showing an increasing trend [135].

Detection of ADEs is one of the main tasks in the pharmaceutical industry, where monitoring drug side effects is a crucial task for pharmaceutical companies developing drugs and the Food and Drug Administration (FDA). Such adverse effects impose substantial clinical and economic burdens and, in severe cases, necessitate post-marketing regulatory action up to and including market withdrawal [54]. Different ADEs are identified during clinical trials via the analysis of discharge summaries. However, they affect only patients who have participated in the clinical trial [102]. Moreover, healthcare providers are limiting reports to serious events only. The majority of people experiencing ADEs are reluctant to report their symptoms through official reporting systems for various reasons, including unfamiliarity with the reporting systems (e.g., the Yellow Card system in the UK^4^). They might also find it difficult to understand the terminology used in those systems or can be unaware of the importance of reporting ADEs [14].

Because of the existing gap between healthcare professionals and the general public (patients) in expressing the same health concepts [60], an alternative approach for detecting ADEs promptly on a larger scale is to analyse social media which is used by billions of people (around 4.7 billion people) around the world [14, 131]. Social media platforms such as Twitter, Reddit, Facebook, Instagram, Pinterest, etc. have been extensively used for market analysis of various products, including medications. Among large volumes of patient-generated content, drugs and ADEs are the most widely discussed topics [81].

Social media has great benefits for detecting ADEs. However, working on data extracted from social media has a set of challenges and limitations, including detecting variations of medical terms, typos, ungrammatical sentences, abbreviations, consumer vocabularies and short forms [126]. In most cases, social media data have to be pre-processed to be used. This includes removing URLs, lower-casing, reducing character elongation and tokenisation [80, 138]. Finally, data collected from social media do not represent the whole population evenly [92]. For example, nearly 60% of Twitter users are aged between 18 and 44, making the collected information highly imbalanced compared to the other patients’ ages [65].

## Adverse Drug Events in Social Media: Related Works

### Methodology

For this literature review, we used Google Scholar^5^ as the primary source to collect studies detecting/analysing adverse events from social media, noting that it indexes the majority of relevant works also retrieved by databases such as IEEE Xplore and Scopus. We employed title-restricted queries, including “social media” AND “adverse drug events”, “social media” AND “adverse drug reactions”, “social media” AND “side effects”, and “social media” AND “adverse reactions”. We then filtered for recent publications (2017 onward). Eligible items were English-language research papers published in conferences or journals; patents and protocols were excluded. We focused on studies proposing NLP or Artificial Intelligence approaches for detecting/analysing ADEs from social media.

Initially, 130 papers were identified. Of these, 6 protocols were excluded. A further 24 items were removed because the full text could not be located or they were conference abstracts without methodological detail (i.e., no description of approaches or experiments). We additionally excluded papers that did not propose approaches for detecting/analysing ADEs from social media. After screening, 100 research papers were retained for analysis. To reduce selection bias, initial screening was conducted by one reviewer, and full-text eligibility assessment by a second reviewer.

The published work on ADE detection in social media can be grouped into the following categories: classification, extraction, normalisation, corpus creation, and other analyses related to ADEs (e.g., drug-ADE correlation studies or sentiment analysis). We present the related work per category in the remainder of this section.

### ADE Classification

This task entails assigning a class label to social media posts (e.g., tweets, forum messages, and comments). In most studies it is formulated as a binary problem with two labels: ADE (texts containing an adverse drug event) and NoADE (texts without an adverse drug event). Classification typically serves as the initial screening stage to determine whether a text references an ADE prior to downstream extraction or normalisation. The following works focus on this task: [2, 16, 23, 29, 37, 48, 54, 63, 64, 80, 87, 100, 102, 105, 125].

A common general system pipeline was used by these authors including five main components:

- Data collection which was mainly conducted using the Twitter API.^6^ Almost all of these studies used Twitter data, with datasets ranging in size between 4,252 tweets [102] and 18,000 tweets [2, 63, 100] and which are in the context of the task dedicated to the classification, extraction and normalisation of Adverse Effect mentions in English tweets as part of the shared task Social Media Mining for Health Applications 2021 (SMM4H). Works on extraction and normalisation will be presented more specifically in the following sections. However, two works focused on other social media where they respectively extracted 261,464 posts from MedHelp^7^ [80] and 10,000 posts from cancer discussion forums [16]. Another work by [48] relied on topic modelling for collecting and filtering tweets allowing for the collection of more than 800,000 tweets during two phases (400,000 during the first phase and 411,010 tweets during the second phase). This work is dedicated to vaccine adverse events, and the tweets were collected using a set of keywords including *vaccination, vaccinations, vaccine, vaccines, vax, vaxx, vaxine, vaccinated, vaccinated, flushot, flu shot*. In addition to the previously cited studies, more recent works have expanded data collection practices in notable ways. [29] gathered data from a variety of online platforms, including Twitter, online forums, and patient review websites, compiling a dataset that includes drug names, associated conditions, and user-provided ratings. [64] utilised an existing annotated Twitter corpus, emphasising the reuse and benchmarking of previously validated datasets (in [23]). Ref. [37] combined Twitter data with PubMed abstracts to examine ADE-related content across both social and biomedical publication platforms.
- Data pre-processing where almost all the authors performed some type of pre-processing of the datasets including tokenisation [23, 80, 105], de-emojisation of tweets, i.e. replacing emojis with their text strings [63, 100] and removal (or replacement) of URLs and other special characters (used by almost all the works).
- Feature extraction mainly using bag-of-word algorithms [80, 87] with the work of [87] also relying on Dimensionality Reduction using PCA, Principal Component Analysis [1]), on the n-gram model [23] or both n-gram analysis and a lexicon [54] or using TF-IDF [37].
- Handling an imbalanced dataset where some works relied on oversampling methods which consisted of duplicating examples in the minority class or synthesizing new examples from the examples in the minority class. The authors relied on different techniques for oversampling the data including WESMOTE, word embedding-based synthetic minority over-sampling technique [23] or a semantic enrichment technique [102], random oversampling combined with an increase of the class weights [2]
- Classification where different machine learning algorithms and embedding models were used, mainly including Support Vector Machine (SVM) [54, 80, 102, 125] and transformer models [129] such as BERT-base [27], BERT-large [27], DistilBERT [111], ALBERT [70], Bio-ClinicalBERT [5], BERTweet [94], XLNET [133], RoBERTa [82]. The latter studies include [2, 63, 100] and [48]. In more recent work, [29] introduced a hybrid classical-quantum model that combines BioBERT with quantum variational circuits, achieving high performance on ADE classification tasks using diverse patient review data. [64] applied ensemble learning by stacking CNN, LSTM, and SVM models with GloVe embeddings, reporting competitive results on an existing annotated Twitter dataset. Additionally, [37] performed a comparative evaluation of multiple machine learning classifiers (including Naive Bayes, SVM, and XGBoost) using TF-IDF features over Twitter and PubMed data, highlighting the generalisability of models across different data sources (Table 1).

**Table 1:** Synthesis of the works on classification

Work	Year	Approach	Social media source	Models (or tools)	Datasets	Best results	Annotation guideline
[80]	2019	A feature-weighted-based improved disagreement-based semi-supervised learning method (WIDSSL)	MedHelp	Random Subspace (RS) method, WIDSSL method, Random Forest (RF), SVM	Collected: 261,464 posts. Annotated: 319 ADEs and 981 NoADEs	AUC: 84.21% (WIDSSL)	No
[105]	2021	To create a labelled database and ontology to improve the pre-processing of tweets for classification	Twitter	Not included	Collected: 30,000 tweets. Annotated: 1,000 tweets	5% of tweets classified as ADEs	No
[23]	2019	A novel word embedding-based synthetic minority over-sampling technique (WESMOTE)	Twitter	AKNN, SMOTE, WESMOTE, WSVM, RUS, VUE, RUSB	Two annotated corpora: PSB (15,717 tweets), SMM (25,678 tweets)	F1-score: 0.426 (PSB-SMM) with RUS_WESMOTE; 0.422 (SMMH) with VUE	No
[16]	2021	Determine the frequency of reportable AEs in a large sample of patient posts	A cancer discussion forum	No models used	Collected: 10,000 posts	AUC: 0.928	No
[102]	2021	A multichannel approach extended to Convolutional Neural Network (CNN)	Twitter	CNN, SVM-MCNN	4,252 annotated tweets	Precision: 0.9, Recall: 0.78, F1-score: 0.82	No
[2]	2021	The use of Bert for the classification	Twitter	DistilBERT, ALBERT, BERT-base/large, Bio-ClinicalBERT, BERTweet, BERTweet-Covid195	17,344 tweets for training, 913 for validation	F1: 84.30 (BERTweet-Covid195)	No
[63]	2021	The use of Bert for the classification	Twitter	BERT, RoBERTa, BERTweet	Training: 18,000 tweets, Validation: 953, Test: 10,000	F1-score: 0.836 (BERTweet)	No
[87]	2017	To train SVM classifier to identify side effects	Twitter	SVM	Dataset includes 7,000 tweets (based on [40])	ACC: 84.21%	No
[54]	2021	Use sentiment features to detect drug-caused side effects	Twitter	NB, LGR, SVM, SGD, kNN, DT, RF, Ensembles	Collected: 486,689 tweets; Final: 226,834 tweets	ACC: 0.776 (Ensembles)	No
[100]	2021	Evaluate transformer-based models with SMOTE and augmentation	Twitter	BERT, DistilBert, XLNet, RoBERTa	Train: 18,000, Validation: 953, Test: 10,000 tweets	F1-score: 0.8433 (RoBERTa + augmentation)	No
[125]	2018	Combine Twitter and VAERS to identify potential AEs after flu shots	Twitter	LibShortText, SVM, LR, NN, miFV, miVLAD, MILR	Twitter (11.9B tweets), VAERS (2500 records)	ACC: 0.86 (MILR)	No
[48]	2022	Topic modeling and classification for vaccine discussions	Twitter	Various including SVM, CNN, BiLSTM, RoBERTa, XLNet	811,000 tweets collected over 2 years	F1-score: 0.919 (RoBERTa Large)	No
[29]	2024	A hybrid classical-quantum model detects ADEs combining machine learning and quantum computing	Online forums, Twitter, patient reviews	Bio-BERT, Quantum Variational Circuit (VQC)	Review datasets with drug names, conditions, and ratings from patient reviews	Accuracy: 97%, F1-score: 97%, Training Loss: 0.0659, Validation Loss: 0.072	No
[64]	2024	Applies ensemble learning (CNN, LSTM, SVM) to detect adverse drug events	Twitter	CNN, LSTM, SVM (base models), Logistic Regression (meta-model), GloVe, vaderSentiment	Twitter ADE dataset (Dai & Wang, 2019)	Stacking (CNN, LSTM, SVM) F1-score: 0.87, Accuracy: 0.89, AUC: 0.91	No
[37]	2023	Compares machine learning methods for binary ADE classification with TF-IDF features	Twitter + Pubmed	Naive Bayes, SVC, LR, RF, XGBoost, AdaBoost, Voting, Bagging, Decision Tree	TwiMed (PubMed), CADEC, ADE: 1644 abstracts with labeled ADR sentences	Naive Bayes: F1-scores - 64.93% (TwiMed), 94.29% (CADEC), 78.76% (ADE)	No

### ADEs Detection

Accurate and timely extraction of adverse drug events (ADEs) from user-generated text is pivotal for pharmacovigilance. Yet pre-approval clinical trials–constrained by limited duration and sample size–capture only a fraction of potential adverse effects, leaving many to be identified post-marketing [81]. A substantial body of work indicates that social media provides complementary early signals for ADE extraction. Realising this potential requires rigorously designed NLP pipelines and alignment with regulatory pharmacovigilance practices, including robust methodology, high-quality annotation, and reproducible evaluation, to support product-safety surveillance. [131].

Two common approaches have been used for medical entity extraction in general: (1) lexicon-based and (2) machine learning-based methods [131]. Some studies adopted the lexicon-based approach and explored the use of existing knowledge bases or customized lexicons, such as United Medical Language System (UMLS), FDA Adverse Event Reporting System (FAERS),^8^ Consumer Health Vocabulary (CHV),^9^ GATE^10^ and Medical Language Extraction and Encoding System (MedLEE) to detect adverse event mentions [10, 58, 80, 122] [96]. However, the majority of the most recent studies rely on machine learning approaches, which usually achieve higher precision and overcome some of the shortcomings and limitations associated with traditional non-learning-based approaches. They include [4, 9, 14, 26, 28, 33, 39, 67, 72, 79, 83, 102, 113, 115, 118, 119, 124, 127, 128, 131, 138, 139].

We also observed that some approaches cannot be classified into those two categories where the authors extracted ADEs from a corpus that was manually annotated without using any lexicon or machine learning techniques [3]. Other approaches exploited various lexical, semantic, and syntactic features, and integrated ensemble learning and semi-supervised learning to detect ADEs [81]. Some authors started by training the embedding model which they used subsequently for the detection. For example, [51] trained and tested AC-SPASM, a Bayesian model for the authenticity and credibility-aware detection of potential ADEs in social media. Finally, in addition to detecting ADEs, some approaches also highlighted the correlation between drugs and ADEs [25]. Ref. [34] builds a knowledge graph of ADEs from Reddit using GPT-4o and visualizes the structure using D3.js. Ref. [108] integrates multimodal data (text and medical images) for ADE detection using vision-language models such as nstructBLIP, GIT, and LSTM with CNN backbones (VGG16 and ResNet50) (Table 2).

**Table 2:** Synthesis of the works on detection

Work	Year	Approach	Social media	Models (or tools)	Datasets	Best results	Annotation guideline
[58]	2021	Analysed the frequency of occurrence of selected common symptoms in Poland	Twitter	No models were mentioned	43,375 Tweets in Polish with #szczepionka. 1,249 reports from postmarketing registry	Pains were the ADEs with the highest frequency	No
[122]	2019	Reporting the occurrence of ADEs when taking medicinal products	Forum (puls.bg)	No machine learning	3,018 user posts	60 ADEs reported	No
[39]	2020	Develop an ADE recognition system and identify potential factors influencing the transferability	Twitter	Bayesian probabilistic model, LR, word2vec	196,533 (138,885 after preprocessing) + 57,473 annotated tweets	F1-score: 0.26 (WEB-RADR reference)	No
[124]	2021	Identify the ADEs associated with kratom and their predominance using social media analytics and data mining techniques	Reddit and Twitter	LDA algorithm, ReadMe, TF-IDF	36,516 posts, 96.8% from Reddit	26% of users’ posts discussed multiple kratom side effects	No
[127]	2021	The use of concept and relation detection to extract Dietary Supplement Adverse Events	Twitter	BERT, CRF, RoBERTa, BioELECTRA, DeBERTa, etc	247,807 tweets; 2,000 manually annotated	F1: 0.866 (concept), F1: 0.788 (relation)	Yes
[72]	2021	A semi-supervised approach estimating ADE severity using social media embeddings	openFDA website	RedMed embeddings, k-NN, node2vec variant	2929 ADEs, FDA AE reports	SAEDR: 0.595, 0.633, $-$0.748 for outcomes	No
[10]	2022	VAERS reports of potential COVID vaccine-associated haematological AEs identified	Twitter	No ML	21 Twitter reports + various AE mentions	Vaginal/menstrual bleeding, miscarriages, clotting events	No
[25]	2020	Adopts Fuzzy Formal Concept Analysis (Fuzzy FCA)	Twitter	Stanford CoreNLP, Fuzzy framework	20k tweets + 4k citing papers	91% of extracted correlations considered reliable	No
[81]	2017	Framework for ADE relation extraction with ensemble and semi-supervised learning	MedHelp	AdaBoost, RS, OpenNlp, SVM	261,464 posts; 1,281 annotated (493 events, 2983 rels)	AUC: up to 81.48% (full feature set)	No
[115]	2020	Detect ADEs in Twitter using a graph-boosted framework	Twitter	GloVe, CNN, Seq2seq-Attn, SDNE, FastText, RNN	608 samples (234 pos, 374 neg)	F1: 74%, +2.9% (multi-channel CNN)	No
[113]	2021	Graph adversary representation (GAR) combining graph embedding and adversarial training	Twitter (TwiMed + TwitterADR)	DeepWalk, Node2vec, CNN, BiLSTM, Attention	TwiMed and TwitterADR datasets	F1: 75.25% on TwiMed	No
[131]	2018	Mining e-cigarette AEs in social media using Bi-LSTM	testbed	Bi-LSTM, CRF, Skip-gram, RNN, MetMap	6 M+ posts from 197k users across 64 brands	F1: 92.9%, Precision: 94.1%, Recall: 91.8%	Yes
[51]	2018	Bayesian model for authenticity and credibility-aware ADE detection	Twitter	AC-SPASM	1.19M tweets from 13,178 users	F1: 80%, Precision@10: 90%	No
[139]	2020	Investigate details of ADE words for better classification performance	Twitter + DailyStrength	SVM (BoW, max/mean pooling)	5076 (Diego lab), 3705 (DailyStrength)	AUC: 94.44%, 88.97%	No
[138]	2021	Adversarial transfer learning for ADEs + PubMed biomedical info	Twitter + PubMed	Adv. transfer learning, charCNN, BiLSTM, attention	TwiMed-PubMed + ADE dataset from 644 PubMed abstracts	F1: 68.58% (Bi-LSTM)	No
[3]	2020	Investigate Instagram content related to acne drug isotretinoin	Instagram	Binary classifier (not part of study)	Public posts between Feb-May 2018	7,661 Instagram posts analyzed	No
[102]	2021	Method to improve Twitter AE identification accuracy	Twitter (SMM datasets)	Multi-channel CNN, SVM	SMM + benchmark datasets	Accuracy: 90%, F1: 82%, Recall: 75%	No
[14]	2018	Determine causal relation between drug and ADE using context	Twitter + Facebook	Linear kernel SVM	Posts with 1 drug/event mention	Accuracy: 77.7% (skip-gram features)	No
[96]	2023	Uses lexicon and semantic type filtering for extracting ADRs in diabetes drugs	AskAPatient, WebMD, Iodine	MetaMap for NER, semantic analysis, interfacing with UMLS	6797 drug reviews across 49 diabetes drugs	2572 ADRs detected, including previously unknown ADRs; 684 unique ADRs identified	No
[28]	2024	A quantum transformer model encodes drug reviews for ADE detection via zero-shot classification	Twitter, Online Forums	Quantum Transformer, Variational Quantum Circuits, Zero-shot Classifier	Public reviews dataset	Accuracy: 93%, F1-score: 0.90	No
[33]	2024	BERT model fine-tuned for ADE extraction with external validation using ADE-Corpus-V2	Twitter	BERT-based, BioBERT, SciBERT	ADE-Corpus-V2, SMM4H	F1 scores: 0.8575, 0.9049, 0.9813 (internal eval); 0.8127, 0.8068, 0.9790 (external eval)	No
[79]	2023	Fine-tunes BioBERT and GPT for ADE classification on social media posts	Twitter	BioBERT-Base, RoBERTa-Base, GPT$-$3.5	MedTxt-SM	F1 scores: 0.91 (BioBERT-Base), 0.90 (RoBERTa-Large)	No
[119]	2023	Integrates VADER sentiment analysis with BERT for enhanced ADE detection from tweets	Twitter	BERT, BioBERT, GPT$-$3.5, RoBERTa	SMM4H, ADE-Corpus-V2	F1 scores: 0.76 for BioBERT-Base, 0.78 for RoBERTa-Base, 0.90 for symptom detection in some cases	No
[83]	2025	A question-answering framework with multi-GRU and attention improves ADR detection on tweets	Twitter	Multi-GRU, vMF, Attention Mechanism	PSB2016-Task1, SMM4H2018-Task3 datasets	F1-score: 81.30% on SMM4H2018	No
[118]	2024	A deep convolutional network integrates sentiment, statistics, and medical keywords for ADE detection	Health forums, medication reviews	DCNN	ADE-Corpus-V2, PubMed datasets	F1-score: 97.63%	No
[9]	2024	Uses AI and NER to process social media posts for unreported ADEs	Reddit, Twitter, SIDER	ScispaCy, NER model, GPT$-$3.5	11,185 Twitter posts, 489,529 Reddit posts, 13,491 PubMed articles, SIDER database	Identified 134 ADEs of GLP-1 Receptor Agonists, including both established and novel ADEs, with clusters and co-occurrences highlighted	No
[4]	2024	Topic modelling and SVM classifier analyse Arabic tweets for vaccine side effects	Twitter	BTM, SVM, Fuzzy String Matching	65,387 tweets (148,324 symptom mentions)	51 symptoms identified; 7 affected systems; clustering of co-occurring symptoms	No
[128]	2025	Combines transformer models with DRUGO ontologies and GAT for ADE detection	Twitter, Ask a Patient, Medical case reports	BERT, BioBERT, ERNIE, GAT	CADEC, SMM4H, PsyTAR, ADE, TAC	F1-scores: 94.15% on TAC corpus with BioBERT and contextual drug knowledge	No
[26]	2024	Fine-tunes BERT, RoBERTa, Bio_ClinicalBERT, and ChatGPT to classify ADEs from Twitter	Twitter	BERT-base, Bio_ClinicalBERT, RoBERTa, RoBERTa-Large, ChatGPT	SMM4H	RoBERTa-Large achieved the best F1-measure (0.80), and ChatGPT fine-tuned performed second best (0.75)	No
[67]	2023	Fine-tunes BERT-based models for ADE extraction from Twitter posts with external validation	Twitter	BERT, RoBERTa, Bio_ClinicalBERT	CADECv2, SMM4H challenge dataset	F1-Score: 0.80 (Achieved by RoBERTa-Large model)	No
[34]	2025	Uses LLMs to extract and structure ADEs from Reddit into a knowledge graph	Reddit	GPT-4o mini, D3.js	Reddit, FAERS	Side effects like nausea, depression, weight gain identified; results validated with FAERS	No
[108]	2024	Integrates textual descriptions and medical images for ADE detection using vision-language models	Twitter, Healthcare blogs	nstructBLIP, BLIP, GIT, LSTM+VGG16, LSTM+ResNet50	MMADE dataset (1,500 image-text pairs)	Best rouge(0.571), Bleu (0.319), BERTScore(0.893), MoverScore(0.6222)	Yes (short)

The aforementioned studies generally rely on a detection pipeline including five main phases:

- **Data extraction**. For extracting the data, two different techniques were used. Some authors collected data from Twitter, Reddit, Facebook or Instagram [3, 9, 10, 14, 25, 26, 58, 79, 119, 124, 127]. While others used other data sources such as PubMed, Europe PMC services, MedHelp or patient forums [25, 34, 81, 96, 113]. The size of datasets varied from hundreds to millions of posts collected over many years [51, 67, 115, 128, 131].
- **Data pre-processing**. Most studies applied common text normalization techniques such as lowercasing, removing numbers and URLs, filtering stop words, segmenting hashtags, and using TF-IDF representations [51, 58, 79, 83, 124, 127, 139]. Some also incorporated fuzzy string matching or language filtering [4].
- **Feature extraction**. Different techniques were explored including lexical and POS (part-of-speech) techniques [10, 81, 96], sentiment-based features [118, 119], graph embeddings and node features [113, 128], and multimodal (text-image) integration [108].
- **Annotation**. Some of the work collecting data from social media relied on manual annotation to improve the training results or for constructing a gold dataset [3, 113]. To improve the quality of the annotation, some authors [108, 127] designed a tailor-made annotation guideline or by using widely accepted corpora such as SMM4H and ADE-Corpus-V2 [33, 67].
- **Named Entity Recognition(NER)**. Different techniques and algorithms were used, including deep neural networks, Bayesian or BERT models, a CRF Classifier, RoBERTa, BioELECTRA [101], DeBERTa [49], the RedMed [71] word-embedding model as well as an SVM [21] model [72, 81, 127]. These were used in various combinations across studies [9, 33, 67, 72, 81, 127, 128].

In addition to ADE extraction, the work published by [81] also focused on extracting the relationship between drugs and ADEs.

### Normalisation

Normalisation refers to mapping extracted adverse drug event (ADE) mentions to controlled vocabulary codes in biomedical ontologies such as the Unified Medical Language System (UMLS), SNOMED CT, and the Medical Dictionary for Regulatory Activities (MedDRA). In most pipelines, normalisation follows detection/extraction: ADE spans are first identified automatically and then linked to ontology entries. To the best of our knowledge, no publications address normalisation as a stand-alone task independent of ADE detection [59]. For instance, [59] employed a neural transition-based named entity recognition (NER) model to extract ADE mentions and subsequently linked each to a MedDRA code, experimenting with GloVe [98], ELMo [99], and convolutional neural network (CNN) architectures [66]. The preprocessing pipeline included whitespace- and punctuation-based tokenisation; lowercasing; replacing URLs with httpurl; replacing user handles with username; and normalising HTML escape characters (e.g.,& ! &).

More recently, [104] proposed a new approach leveraging the BioLORD model and its variants, including BioLORD-STAMB2 and BioLORD-STAMB2-STS2, for the normalisation of ADE mentions in social media. Their system was evaluated on several benchmark datasets such as CADEC, PsyTAR, and TwiMed, and showed significantly improved performance. It achieved F1-scores of 60.28 for CADEC, 65.49 for PsyTAR, and 50.57 for TwiMed using the BioLORD-STAMB2-STS2 variant. These results highlight the effectiveness of sentence-transformer-based biomedical representations, particularly when fine-tuned with semantic textual similarity tasks, for ADE normalisation (Table 3).

**Table 3:** Synthesis of the works on normalisation

Work	Year	Approach	Social media	Models(or tools)	Datasets	Best results	Annotation guideline
[59]	2021	Recognize the adverse drug effect (ADE) mentions from tweets and normalize the identified mentions to their mapping MedDRA preferred term IDs	Twitter	Glove, ELMo, Neural Transition-based Model for named entity recognition (NER), CNN	29,274 tweets and MedDRA v21.1 KB with 25,463 unique preferred term IDs	F1: 0.220, R: 0.218, P: 0.231 (for the normalisation with neural transition-based joint mode)	No
[104]	2023	Uses BioLORD model with STS fine-tuning for ADE normalisation in social media	Twitter	BioLORD, BioLORD-STAMB2, BioLORD-STAMB2-STS2	CADEC, PsyTAR, TwiMed, SMM4H	F1 scores: 60.28 for CADEC, 65.49 for PsyTAR, 50.57 for TwiMed (BIOLORD-STAMB2-STS2)	No

### Resource Creation

As noted in Sects. 4.2 and 4.3, machine-learning approaches predominate for automated ADE detection. However, these methods require substantial volumes of expertly annotated data, making corpus construction costly and time-consuming [115]. Consequently, several studies have focused on developing benchmark resources [6, 30, 62, 68]. Both [30] and [6] created reference datasets for evaluating system performance: [30] curated and manually annotated a Twitter corpus of 57,473 de-duplicated, sampled tweets^11^ , while [6] produced the TwiMed dataset comprising 1,000 annotated tweets and 1,000 PubMed sentences focused on ADEs related to *Diclofenac* and *Lipitor*. Other efforts have leveraged drug names as retrieval keywords to assemble candidate posts, including Twitter data [68] and patient-forum narratives from AskaPatient [62], the latter introducing the CADEC corpus (A Corpus of Adverse Drug Event Annotations)^12^

Other studies focus on validating the constructed corpus either by classifying ADEs [35, 47, 60, 77, 114, 117] or by extracting them [7, 77].

Other studies focus on validating the constructed corpora either by classifying ADEs [35, 47, 60, 77, 114, 117] or by extracting them [7, 77].

For this group of works, Twitter was also the predominant source for collecting data [35, 47, 60, 77, 114, 117]. Ref. [114] also used MedHelp posts and [7] extracted their dataset from French health forums.

More recently, four additional studies contributed notable advances to resource creation. Ref. [112] annotated VAERS reports using MedDRA to enable temporal analysis of vaccine-related adverse events, achieving a significant improvement in inter-annotator agreement from 69% to 86% after refining guidelines. Ref. [88] applied transfer learning models, including BERT and BETO to classify Spanish-language COVID-19 vaccination tweets, with RoBERTuito achieving the best F1-score (0.79), demonstrating strong performance compared to traditional classifiers. Ref. [85]evaluated ADE classification robustness across linguistic factors using handcrafted templates, reporting F1-scores of around 0.70 on held-out test sets for both BioRedditBERT and XLM-RoBERTa. Finally, [24] introduced the MultiADE benchmark for ADE extraction across heterogeneous sources such as clinical notes, scholarly articles, and social media, reporting the highest F1-score of 69.0% using RoBERTa-Large in its domain.

Several corpora were released alongside complementary resources and tools for processing, including ADRMine (a conditional random field-based sequence-labelling system for ADE extraction) [117], UMLS [77, 117], cTAKES for clinical concept extraction [60], and the FDA Adverse Event Reporting System (FAERS) [77]. Across this body of work, a wide range of classifiers and sequence models were explored–from traditional baselines (logistic regression, LR [22]; stochastic gradient descent, SGD, classifiers [106]; linear SVC) to neural architectures (multi-channel CNNs [47, 114]; LSTM [52]; GRU [19]; BiGRU [18]; CNN–BiLSTM; CNN–BiGRU) and large pretrained transformers (BERT; RoBERTa/RoBERTa-Large; XLNet/XLNet-Large; XLM) [47, 69], as well as Bayesian hierarchical models [77] (Table 4).

**Table 4:** Synthesis of the works on resource creation/ resource creation + classification/ resource creation + extraction

Work	Year	Task	Approach	Social media	Models (or tools)	Datasets	Best results	Annotation guideline
[30]	2020	Resource creation	Create a benchmark dataset to evaluate ADE recognition systems	Twitter	No model used	5.6M tweets ? 57,473 after sampling	1,056 ADEs, 56,417 NoADEs	Yes
[68]	2018	Resource creation	Use keyword combinations and filtering for data collection	Twitter	No model used	10,000 keywords ? 438 (scenario1), 1,323 (scenario2) tweets	Tweet counts per scenario	No
[6]	2017	Resource creation	Benchmark corpus to compare drug reports in Twitter vs. PubMed	Twitter	Brat and Knowtator (annotation)	29,435 PubMed + 165,489 Twitter sentences	3144 entities, 2749 relations, 5003 attributes	Yes
[62]	2015	Resource creation	Construct CADEC corpus for ADEs in medical forums	AskaPatient	No model used	1,253 posts	9,111 entities: 69.3% ADEs, 19.8% drugs	Yes
[112]	2024	Resource creation	Annotates VAERS reports using MedDRA for temporal analysis of vaccine-related events	VAERS reports	MedDRA terms	282 VAERS reports (1990-2016)	Inter-annotator agreement improved from 69% to 86% after refining guidelines	Yes
[117]	2018	Resource creation + classification	Compare ADE mentions in FAERS, DIDs, and Twitter	Twitter	Not mentioned	10,188 tweets (Humira/adalimumab keywords)	Reported ADEs resemble FAERS more than DIDs	No
[114]	2018	Resource creation + classification	NLP + DNN-based method to mine ADEs	Twitter	Skip-gram, CNN, TF-IDF, Word2Vec	1013 ADE, 3122 NoADE samples	F1: 74.4% (multi-channel CNN)	No
[47]	2021	Resource creation + classification	Identify effective NLP pipelines for VAEM tweets	Twitter	Variety: Logistic RCV, SVMs, CNN, RNNs, RoBERTa, BERT, etc	688,357 tweets total	F1: 0.91 (RoBERTa Large)	No
[35]	2019	Resource creation + classification	System to collect/process drug-related tweets for ADEs	Twitter	Not mentioned	TweetAEMiner tool + drug-specific words	Detected 8 known + 2 novel doxycycline AEs	No
[60]	2018	Resource creation + classification	Expand Consumer Health Vocabulary with Twitter terms	Twitter	gensim, word2vec, VSM, SIDER	53 M tweets using 1,147 meds as keywords	333 new side effect terms found (vs. 90 in CHV)	Yes
[77]	2020	Resource creation + extraction	Compare signal detection from Twitter vs. SRS	Twitter	MCEM model, Bayesian hierarchical model	192,000 tweets from 4 datasets	AUC (combo): 0.587$-$0.637; Twitter: 0.525$-$0.534	No
[7]	2018	Resource creation + extraction	Protocol for evaluating ADE extraction tools	atoute, doctissimo, e-sante, aufeminin	NER and entity recognition models	325,535 annotated forum messages	Precision, recall, CI at 95%	Yes
[88]	2024	Resource construction + classification	Uses transfer learning models (BERT, BETO) to classify Spanish post-vaccination tweets	Twitter	BERT, BETO, RoBERTuito, SVM, RF	1332 Spanish tweets related to COVID-19 vaccination	RoBERTuito achieved the best F1 score (0.79), outperforming traditional models	Yes
[85]	2024	Resource construction + classification	Uses handcrafted templates to evaluate ADE classification robustness across linguistic factors	Twitter, Reddit, PsyTAR	BioRedditBERT, XLM-RoBERTa	Custom dataset (SMM4H-2021, SMM4H-2017, NADE)	BioRedditBERT and XLM-RoBERTa achieved similar results on the held-out test set, with F1-scores of around 0.70	No
[24]	2024	Resource creation + extraction	Builds MultiADE benchmark for ADE extraction across clinical notes, scholarly articles, and posts	Existing dataset	RoBERTa, GPT-4, Llama-3, BART	n2c2, MADE, PHEE, PsyTAR, CADEC, CADECv2	RoBERTa-Large achieved the highest F1 score for ADE recognition (69.0%) in its domain	Yes

### Pipelines for Classification, Detection and Normalisation

While some of the previously reviewed work focused on either the classification or the extraction, some studies combined both tasks in a pipeline, e.g. [134]. Many other studies [13, 38, 45, 65, 121, 126, 130, 137], followed the same approach, while others [8, 31, 36, 57, 86, 103, 109, 140, 141], added normalisation to the pipeline to map the extracted ADEs to codes in the commonly used ontologies such as SNOMED-CT or UMLS.

**Fig. 1:**
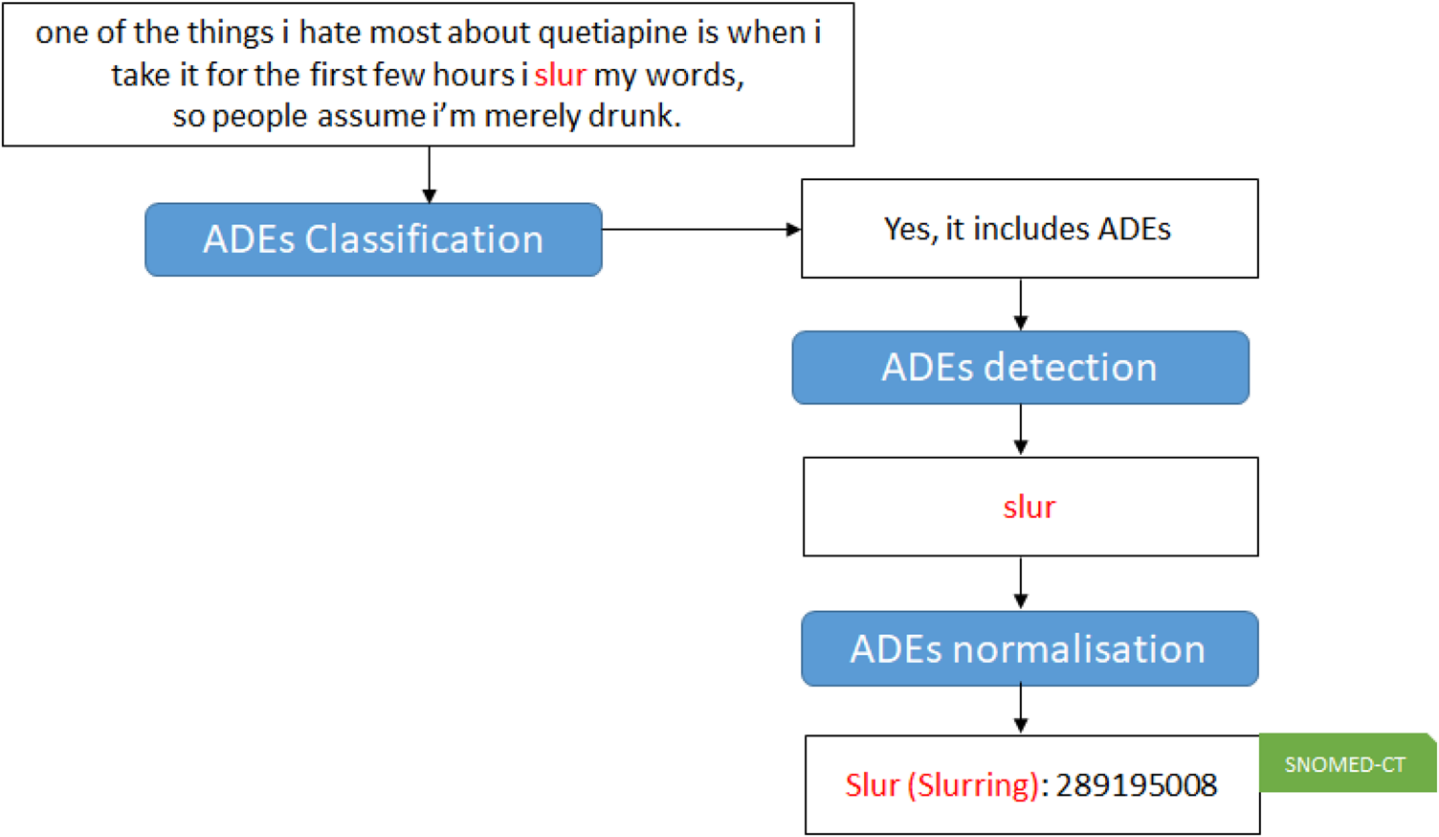
Pipeline steps on an example

The majority of the works employing the entire pipeline (classification, extraction and normalisation) were proposed in the context of the SMM4H shared task where participants could either propose a system classifying ADEs, classifying and extracting ADEs or the entire pipeline where the participants could also link the extracted ADEs to an ontology such as MedRRA [31, 36, 103, 109, 140], (SMM4H 2021) as well as the work of [8] (SMM4H 2019). Others did not participate in the shared tasks but also used the SMM4H dataset [86]. Others used the MADE dataset [57].^13^ In order to highlight the different steps of the pipeline on a proper example, we introduce Fig. 1

All the aforementioned studies rely on a pipeline including five phases:

- **Data collection**: most of the studies focused on data obtained from social media platforms such as Twitter, Facebook, etc. [8, 13, 45, 65, 78, 141]. Some other data sources were also used such as CADEC [109, 121] or other publicly available datasets, including MADE 1.0^14^ [57] and SMM4H^15^ [31, 38, 109]. However, all of these corpora were constructed from social media or forums. Some works also made use of different corpora including TwiMed and TwitterADR, in addition to SMM4H [126] where the authors used 160GB of data collected from BookCorpus,^16^,^17^,^18^ and Stories. The size of the datasets varied among the studies where [8] used 2,367 tweets, [78] used 5,600 tweets, [65] used 34,293 tweets and [31] used 17,385 training samples. Additionally, [130] used FDA drug labeling documents to automate ADE annotation through a Retrieval-Augmented Generation (RAG) mechanism and a large language model (LLM), achieving F1 scores of 0.978 for DILI, 0.931 for DICT, and 0.911 for AE profiling. Additionally, [130] used FDA drug labelling documents to automate ADE annotation through a Retrieval-Augmented Generation (RAG) mechanism and a large language model (LLM), achieving F1 scores of 0.978 for DILI, 0.931 for DICT, and 0.911 for AE profiling.
- **Data preprocessing**: This task is also variable and depends on the quality of the available data. Some works such as [121] did not conduct any pre-processing themselves as they used publicly available corpora which were previously pre-processed such as CADEC. Other works [38, 45, 103, 126] applied the most commonly used pre-processing techniques, including lowercasing, URL removal, non-alphabetic character removal, stop word removal, tokenisation and special character removal.
- **Word embedding and feature extraction**: [65, 126, 38] and [13] considered this to be an essential step in converting text data (letters, words, sentences) into feature vectors that encode the meaning of the text so that instances that are closer in vector space are expected to be similar in meaning. Others used only bag-of-wrords (BOW) and TF-IDF techniques [13, 38].
- **Data annotation**: As mentioned in the previous section, this phase is used to label data collected from social media. For this purpose, [65] labelled their corpus as non-medical or medical methylphenidate^19^ after collecting data from Twitter. Ref. [78] manually annotated their corpus to identify tweets containing personal experiences regarding COVID-19 vaccinations.
- **Classification, extraction and normalisation**: The majority of works relied on transformers for the classification step such as BERT, BERTweet, RoBERTa, BioBERT [74], Bio-clinicalBERT [31, 36, 86, 103, 141], with contextual embeddings such as ELMo [8] were also being used. Glove and FastText [12] were also used by some studies [86]. Some authors applied LSTM, bi-LSTM, CRF [69] or RNN [53] layers for classification [8, 36]. For the extraction step, authors tended to use the same models used for classification only or for both classification and normalisation, e.g. [36, 109] used a neural model combined with BERT for extraction and normalisation. Some other works employed a Named Entity Recognition (NER) pipeline combined with different models such as RoBERTabase and BERTweet for the extraction phase [103]. More recently, [140] used RoBERTa, GPT-4, and BioBERT for ADE classification and normalisation on Twitter, reporting F1 scores of 0.838 (RoBERTa), 0.306 (GPT-4), and 0.354 (BioBERT).

In this section, we provided an overview of studies that employed a pipeline-based approach using classification and extraction as well as studies which added the normalisation step, resulting in high performance and very good results in the majority of cases (Table 5).

**Table 5:** Synthesis of the works on pipelines: classification + extraction / classification + extraction + normalisation

Work	Year	Task	Approach	Social media	Models (or tools)	Datasets	Best results	Annotation guideline
[134]	2021	Classification + extraction	Task 1 (classification and extraction of ADEs) of the shared task SMM4H 2021	Twitter	SVM, RBF, BERT variants, BiLSTM-CRF, fastText, BytePair	17,385 tweets (classification), 1,717 tweets (extraction)	F-score: 0.46 (classification), 0.50 (extraction)	No
[38]	2022	Classification + extraction	Sentence pair classification with BERT	Twitter	BERT, RoBERTaLARGE	29,529 SMM4H + 160GB data	F1: 0.64 (RoBERTa)	No
[126]	2017	Classification + extraction	Quantum Bi-LSTM with attention for ADE detection	Twitter	QBi-LSTMA, Bi-LSTM, LNS, CNN, RNN	TwiMed (1,000), TwitterADR (10,822)	F1: 73.62% (QBi-LSTMA)	No
[45]	2021	Classification + extraction	Two separate systems using Transformer models (SMM4H 2021)	Twitter	BERTweet, BioBERT, SciBERT, RoBERTa	28k (classification), 18,300 (extraction)	F1: 40.0 (classification), 47.3 (extraction)	No
[137]	2019	Classification + extraction	Lexicon-based ADE extraction + binary classification	Chinese social media	SVM, HMM, CRF, pattern-based classifier	456,753 messages ? 302,180 sentences	Accuracy: 83.1% (SVM)	No
[13]	2018	Classification + extraction	Causality measure for ADEs based on classification	Twitter, Facebook	SVM, BOW, RBF, CNN	44,809 positive + 50,081 negative instances	Accuracy: 74% (BOW)	No
[121]	2018	Classification + extraction	Detect ADE spans using LSTM-CRF model	Twitter, Facebook	LSTM-CRF	CADEC + 1250 forum posts	F1: 69.94%, P: 68.82%	No
[65]	2020	Classification + extraction	ML analysis of tweets about methylphenidate	Twitter	SVM	34,293 tweets	F1: 0.733, P: 0.920, R: 0.609	Yes
[78]	2022	Classification + extraction	ML pipeline to identify COVID-19 vaccine experiences	Twitter	SVM, Logistic Regression, RF, CRF, etc	111,229 tweets	Best: random forest	No
[130]	2025	Classification + extraction	Automates ADE annotation with RAG mechanism and LLM using FDA labelling documents	FDA drug labeling documents	AskFDALabel (LLM-powered), Retrieval-Augmented Generation (RAG)	DILI (287 annotated drugs), DICT (1167 labeled drugs), AE profiling (200 drugs)	AskFDALabel achieved F1-scores of 0.978 for DILI, 0.931 for DICT, and 0.911 for AE profiling, outperforming traditional methods	No
[93]	2024	Classification + extraction	A two-level Bi-LSTM classifier filters and contextualises ADR mentions in tweets	Twitter	Bi-LSTM (2 levels), BioBERT, SNScrape	499,031 tweets (Covaxin + Covishield, Jan-Dec 2021)	F1-score: 94.33%	No
[141]	2021	Classification + extraction + normalisation	ADE classification, span extraction and normalisation	Twitter	BERTweet, RoBERTa, BERT variants	29,284 English tweets	F1: 0.49 (class), 0.42 (extract), 0.28 (norm.)	No
[36]	2021	Classification + extraction + normalisation	Joint training approach for ADE classification/extraction/normalisation (SMM4H 2021)	Twitter	BERT, Bi-LSTM	25,870 tweets + CADEC	F1: 70.1 (class), 37.0 (extract), 50.3 (norm.)	No
[86]	2021	Classification + extraction + normalisation	ADE classification, span detection, and normalisation	Twitter	BERT, Glove, FastText, TwitterHealth	29,284 tweets with 2,765 ADE mentions	F1: 0.319 (BERT)	Yes
[57]	2020	Classification + extraction + normalisation	MADE 2018 challenge overview on ADEs from EHRs	EHR notes	LSTM, CRF, SVM, RF	MADE 1.0 corpus	F1: 0.8527 (NER), 0.8777 (RI), 0.6612 (NER-RI)	Yes
[109]	2021	Classification + extraction + normalisation	ADE detection with cross-lingual BERT-based models	Twitter	RoBERTaLarge, EnRuDR-BERT, ChemBERTa, HuggingFace models	29,283 English + 20,704 Russian tweets	F1: 0.61 (class), 0.40 (extract), 0.29 (norm.)	No
[31]	2021	Classification + extraction + normalisation	Multi-task learning with transformer models (SMM4H 2021)	Twitter	BERTbase, BioBERT, Bio-ClinicalBERT	17,385 training samples, 23k MedDRA terms	F1: 63.5 (class), 56.0 (extract), 18.5 (norm.)	No
[103]	2021	Classification + extraction + normalisation	ADE pipeline from SMM4H 2021 with multiple BERT models	Twitter	RoBERTa, BERTweet, BioBERT, DEBERTa	18k tweets (class.), 1,234 tweets (extract)	F1: 61% (class), 50% (extract), 94% (task2)	No
[8]	2019	Classification + extraction + normalisation	ADE detection with bi-LSTM and CRF using char + token embeddings	Twitter	bi-LSTM, ELMo, CRF, RNN	3,367 tweets (1,712 positive, 1,655 negative)	F1 (Relaxed): 59.7, Strict: 40.7	No
[140]	2024	classification + extraction + normalisation	Uses RoBERTa, GPT-4, and BioBERT for ADE mention classification and normalisation on Twitter	Twitter	RoBERTa, GPT-4, BioBERT	#SMM4H 2024 Task 1 dataset	F1 scores: 0.838 (RoBERTa), 0.306 (GPT-4), 0.354 (BioBERT)	No

### ADEs Analysis

The last group of publications is centred around an analysis related to ADEs [17, 20, 32, 42, 43, 61, 75, 76, 84, 92, 107, 116, 120, 132, 136, 142]. These analyses involve examination of sentiments, expectations, and anxieties but also ADEs related to vaccine of social media users reporting ADEs [20, 42, 75, 76, 120, 136]. They can also be related to linguistic features validated by clinical experts for detecting ADEs [84] or to a comparison of ADEs related to a given drug with others or evaluating the use of Complementary and Alternative Medicine (CAM) [43, 92, 107]. Finally, some analyses are dedicated to evaluating the precision and accuracy of ADEs reported on social media [142].

Analytical studies extend beyond textual content to incorporate engagement and source metadata (e.g., likes, comments, post type, platform, language, purpose). For example, [75] conducted a retrospective assessment of 600 Twitter and Instagram posts tagged #covidvaccinesideeffects, recording likes, comments, post type, language, purpose, and source; educational quality was independently rated by three examiners with different training levels. Similarly, [20] investigated whether social communication predicts expectations of post-vaccination side effects: in a prospective longitudinal survey, exposure to side-effect information from social media, news reports, and personal acquaintances–and corresponding expectations–was measured pre-vaccination, followed by assessment of experienced side effects post-vaccination.

For this group of studies, the pipeline includes only three phases related to collection, pre-processing and analysis. Similarly, as for previous work, Twitter was the predominant social media source used. Some authors used a list of keywords to extract the tweets automatically. For example, [107] built a list of food and drug administration names, including 297 brand names mapped to 49 generic names. Based on this list, they extracted English posts containing brand names or generic names of these drugs from Twitter.

The work of [132] analysed the different ADEs mentioned on social media. They also presented a synthesis related to similar social media posts involving ADEs and related drugs. They compared the different numbers of mentions related to drugs and ADEs across different social media such as Twitter and Facebook. They concluded by presenting a general percentage associated with the mention of ADEs related to some drugs. For example, they found that 4% of ADEs mentioned in social media are related to a *steroid*, whereas 59% are related to *antibiotics*.

More recently, several studies have been proposed within this area. Ref. [61] conducted a retrospective analysis of Reddit posts over eight years using a Python-based categorisation approach, identifying significant changes in liver and other clinical markers. Ref. [136] used text and graph mining techniques on 386,565 tweets and VAERS data to detect both officially known and previously undocumented COVID-19 vaccine side effects. Ref. [116] evaluated TikTok videos on weight loss medications using the PEMAT-AV tool, finding low overall understandability (43%) and actionability (20%), though personal experience videos scored better. [76] analysed 169 million COVID-19-related tweets using deep learning-based named entity recognition to extract ADE-related sentiments. Finally, [32] compared several biomedical NLP models, including BERT, Sentence-BERT, and SapBERT, for multi-label classification and entity linking on 4,195 Facebook posts from a gastrointestinal disorders forum, reporting improvements in F1 score when using a coarse-grained ontology (Table 6).

**Table 6:** Synthesis of the works on ADEs analysis

Work	Year	Approach	Social media	Models (or tools)	Datasets	Best results	Annotation guideline
[42]	2019	Explore ethical implications of using social media to track adverse events using a multi-method approach	Twitter, Facebook, Instagram	No model used	Open discussion data from August 2018	Some participants opposed social media use for AE research	No
[75]	2022	Analyze posts with #covidvaccinesideeffects by metadata and educational quality	Twitter, Instagram	Statistical analysis	Posts from Jan-Apr 2021 using specific hashtag	Interrater agreement: 89%	No
[84]	2020	Categorize data veracity levels and analyze linguistic features	Twitter	Multinomial Logistic Regression	10,822 annotated tweets, SIDER 4.1	7.98% poor, 43.46% moderate, 48.56% good veracity	Yes
[43]	2020	Compare statin ADEs across social media and regulatory sources	Twitter	No machine learning	HLP DB, FAERS, MHRA, MedDRA, etc	High agreement between Twitter and official sources	No
[20]	2022	Assess how social communication affects expectations and experiences of vaccine side effects	Twitter	Regression model	Pre- and post-vaccination surveys (Apr-Jul 2021)	P = 0.917 (contacts vs. expected side effects)	No
[17]	2018	Study CAM use by AIH patients via AIH-focused Facebook groups	Facebook	No ML	401 user responses with health and CAM data	5% reported serious AEs; 1% hospitalized	No
[107]	2021	Analyze antidepressant side effects from real-world expressions	Twitter	SAGE model	707 M tweets by 283,374 users	Focused on 5 key side effect categories	No
[92]	2022	Analyze ADEs and sentiment for 18 MS drugs on Twitter	Twitter	Crimson Hexagon classifier, ReadMe algorithm	51,362 tweets (2010-2020)	Injectable side effects more prevalent than oral/infusion	No
[142]	2020	Validate social media methodology for detecting pharmaceutical ADEs	Twitter	No models mentioned	40,000 tweets + 40,539 FAERS reports	Only a few common drugs had sufficient ADE content	No
[120]	2018	Identify cluster anxiety-related AEFIs from social media and search data	Facebook	No model used	39 AE reports analyzed	18 cluster events not found in peer-reviewed literature	No
[132]	2021	Detect AEs from posts related to specific medications across platforms	Twitter, Facebook, YouTube, Tumblr, Reddit, etc	No model used	Grey literature from 9 platforms	General detection of AEs across social platforms	No
[61]	2025	A retrospective analysis of Reddit posts over eight years using Python-based categorisation	Reddit	Python-based script	3877 posts from Reddit	Significant changes in liver and other clinical markers	No
[136]	2024	Analyzes vaccine side effects with text mining using Twitter and VAERS data	Twitter, VAERS	Text mining, Graph mining	Twitter (386,565 tweets), VAERS (side effects data from 1990-2021)	Detection of both officially known and unknown vaccine side effects	No
[116]	2024	Assesses TikTok video understandability using the PEMAT-AV tool for weight loss medications	TikTok	PEMAT-AV	Top 50 videos for #ozempicsideeffects, #semaglutidesideeffects, #mounjarosideeffects, #wegovysideeffects	Most videos had low understandability (43%) and actionability (20%), but personal experience videos had higher understandability	No
[76]	2025	Analyzes COVID-19 sentiments using deep learning-based NER for ADE extraction	/	/	Dataset comprising 169,659,956 COVID-19-related tweets from 103,682,686 users. Identification of 2,124,757 relevant tweets	/	No
[32]	2023	Compares NER with entity linking and multi-label classification using Sentence-BERT	Facebook	BERT, PubmedBERT, EndrBERT, Sentence-BERT, BioSyn, SapBERT	4195 posts from 527 discussion threads (GIST forum)	Micro F1: 0.220 (MLC), improved to 0.498 with coarse ontology level	Yes

## Analysis of the Studied Works

In total, we reviewed, analysed and classified 100 research publications. We grouped the papers into six different categories: classification, detection, normalisation, the pipeline including classification, detection and normalisation, resources construction and works presenting an analysis of ADEs. Different social media, forums and other sources were used for collecting data, including Twitter, Facebook, Instagram, AskPatient, MedHelp, PubMed and others.

Different conclusions can be drawn from all of the studies which we reviewed. We present our analysis using the following seven different topics:

1. Tasks involved in the studies
2. Social media source used
3. The proposition of annotation guidelines
4. Embedding models used
5. Machine learning models used
6. Drugs referenced within the studies

### Tasks Iinvolved in the Studies

All the reviewed works can be categorised into one of six main tasks: classification, extraction, normalisation, resource construction, pipeline development, and analysis. Extraction was the most common task, with 32 out of 100 studies (32%) focusing on extracting adverse drug events (ADEs) from social media. Pipeline approaches were also prominent, appearing in 20 studies (20%), often combining multiple tasks such as classification and extraction (11/100; 11%) or integrating classification, extraction, and normalisation (9/100; 9%).

Classification was performed in 15 studies (15%), making it a widely used method across different ADE detection workflows. Similarly, resource construction and analysis were each the focus of 15 and 16 studies, respectively, indicating strong interest in building foundational tools and evaluating ADE trends. Among the resource construction efforts, 9 studies (9%) also validated their resources using classification techniques. Lastly, normalisation was the least addressed task, with only 2 studies (2%) focusing specifically on mapping extracted ADEs to standardised terminologies.

### Social Media Source Used

Twitter was the most used social media platform for extracting data. This is mainly due to the huge amount of data generated daily (500 million tweets) [10, 36, 54, 58]. We can observe that 60 (60%) studies used Twitter data. A subset of those, 11/15 (73.33%) studies, used Twitter data for the classification task, whereas the two other remaining studies respectively focus on MedHelp, Cancer forum, patient reviews and pubmed. Twitter was also predominantly used for normalisation, resource construction and multiple tasks combined in a pipeline where respectively 2/2 (100%), 10/15 (76.66%) and 15/20 (0.75%) studies relied on Twitter. Twitter was used less for the detection and analysis tasks, where respectively 15/32(46.87%) and 6/16(37.5%) studies were on this social media.

**Fig. 2:**
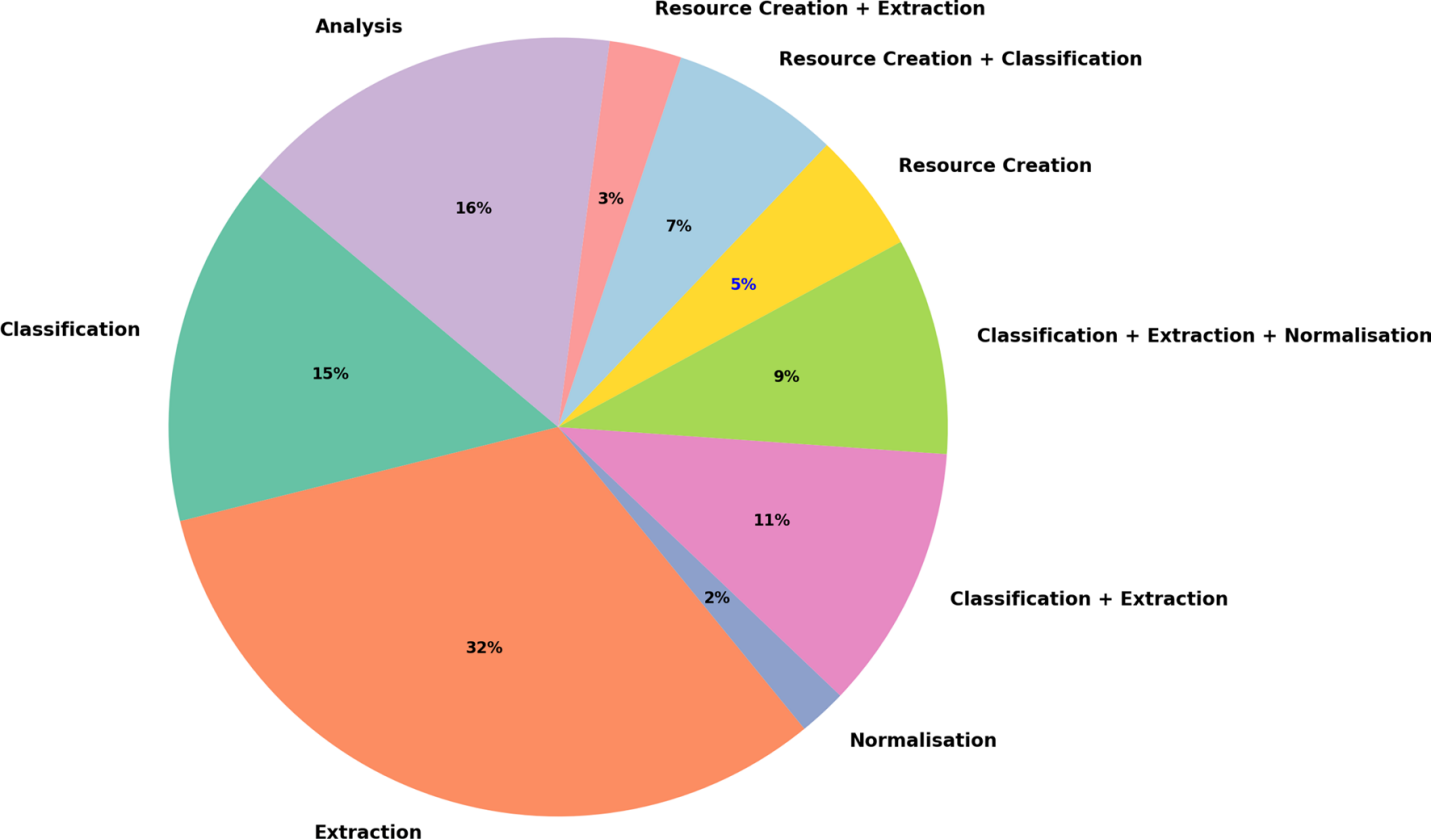
Proportion of studied tasks

Another important aspect to note is that some studies did not exclusively use Twitter data but applied their approaches to data from different social media sources. In these cases (18/100 studies; 18%), Twitter data was used in combination with data from other social media, including Facebook, Instagram, DailyStrength, Reddit, VAERS,^20^ PsyTAR, Healthcare blogs, AskaPatient and PubMed (represented as Twitter + other). Only a few studies were carried out using other social media such as Facebook (3/100; 3%), Instagram (1/100; 1%), Tiktok (1/100; 1%), Reddit (1/100; 1%) and other health social media such as AskPatient (2/100; 2%), MedHelp (2/100; 2%), health forum (2/100; 2%) and BedTest (1/100; 1%). Some works used data from the Food and Drug Administration website^21^ (2/100; 2%), VAERS (1/100; 1%) and Electronic Health Records (EHR) (1/100; 1%). Finally, 5/100 (5%) of the studies were carried out using data from forums and websites in other languages (e.g. Belgian, French, Chinese, Spanish and Arabic).

**Fig. 3:**
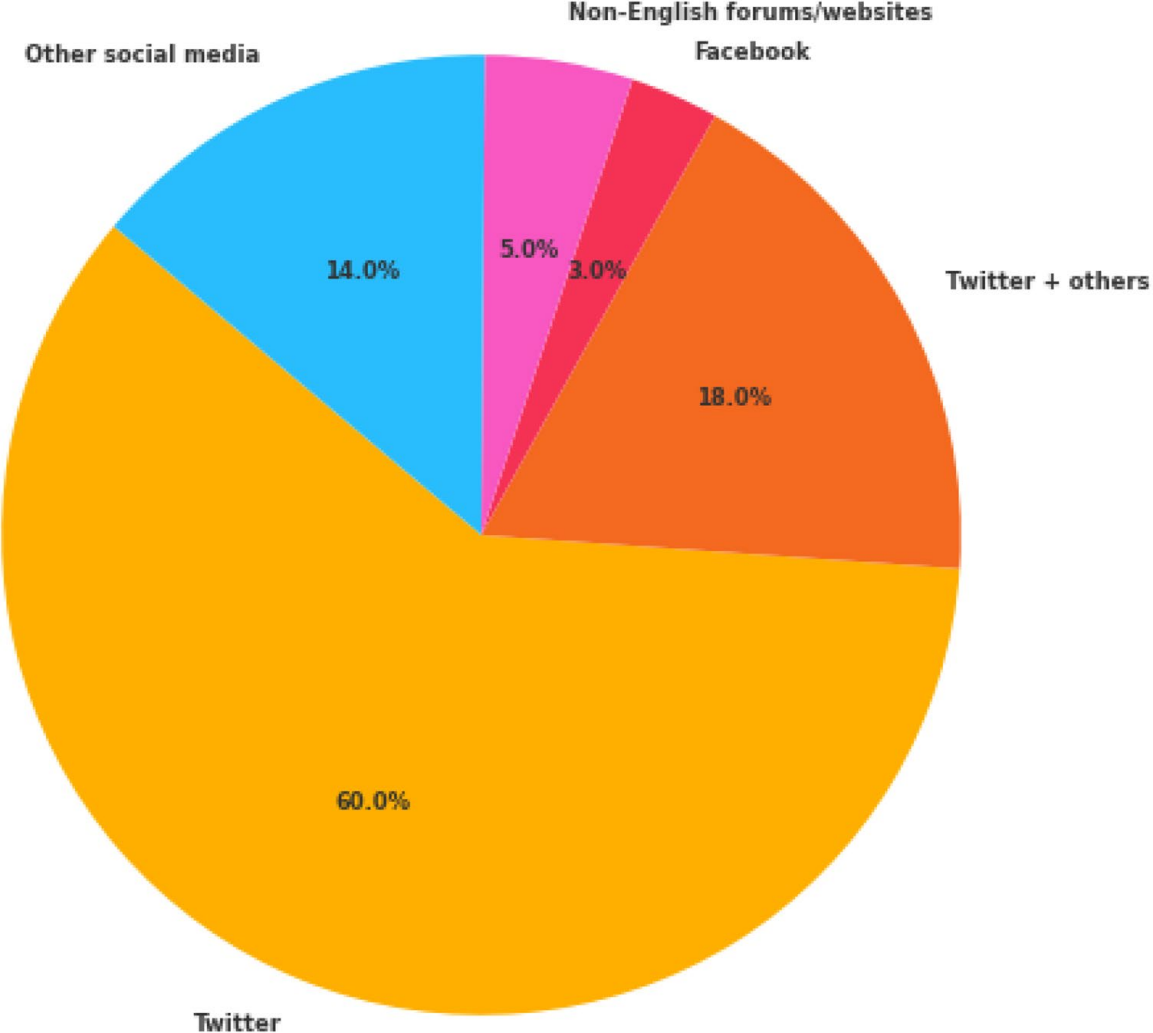
Proportion of social media sources used.

Figures 2 and 3 illustrate the proportion of the different studied tasks as well as the proportions of social media sources used within the studies. In Fig. 2 we have the proportion of all the presented tasks: Classification (i.e. studies detecting if the text includes or not ADEs). Extraction (i.e. studies extracting ADEs from text). Normalisation (i.e. studies mapping the ADEs to an ontology). Classification + Extraction (i.e. studies classifying the ADEs first and extracting them after). Classification + Extraction + Normalisation (i.e. studies classifying ADEs, extracting them and associating them to an ontology). Corpus creation (i.e. studies creating dataset). Corpus creation + classification (i.e. studies creating datasets and validating them by proposing a classification approach). Analysis (i.e. studies analysing ADEs and associating them to other NLP tasks such as sentiment analysis) On Fig. 3 we represent the proportion of all the social media that have been used such as Twitter, Facebook, Instagram, MedHelp, etc. We also have a case where Twitter has been used in addition to other social media such as Facebook, Instagram or DailyStrength. In this scenario, we are using the label Twitter + others on the figure.

### The Proposition of Annotation Guidelines

The training of the different models requires annotated data. The quality of the outputs returned by a model mainly depends on the quality of the annotated data used. Hence, manual annotation is a fastidious task requiring coherence, consistency and precision. In order to provide this precision, an annotation guideline should be prepared before starting the annotation. This guideline is used by the different annotators in order to ensure coherence and consistency. However, from the literature, we observed that only a few works were dedicated to the presentation of annotation guidelines. The scarcity of annotated datasets and the unavailability of annotation guidelines remains one of the most important challenges related to NLP in general. For example, based on our studied papers, we observed that only 12 (12% of the studies) studies proposed an annotation guideline [7, 24, 30, 32, 60, 65, 84, 88, 108, 112, 127, 131].

However, we observed that in the majority of the cases, the authors are providing only a few details regarding the annotation guideline. For example, Xie et al. [131] just mentioned the fact that a random part of the corpus was annotated by two experts independently. They also provide a table highlighting the different annotated entities with an explanation and an example for each entity. However, they also provided the measure inter-annotator reliability (Cohen’s kappa) [11] that was very high with a value of 0.96). [65] also briefly presented their annotation guideline for identifying tweets mentioning first-hand experience by two annotators. These authors have used the Cohen kappa as well. However, they did not provide the value obtained. They mentioned that any disagreements were resolved by discussion among psychiatrists. [60] also briefly present their annotation guideline by mentioning that the two first authors were annotating a list of ADEs concept and their similarity to a list of terms from Twitter. [84] presents an annotation protocol in the form of a flow chart where they are guiding the annotators. Each expert followed this protocol and independently performed the annotation task. The inter-rater reliability was also measured using Cohen’s Kappa (it was 0.80). Disagreement in annotations was resolved during the panel discussion. [127] presents their annotation guideline as supplementary material. The authors were interested in the extraction of the NER and relationships among four entities supplements, drugs, food and health outcomes. The inter-rater agreement (kappa score) for the concept extraction task was 0.9416 and 0.8299 for the relation extraction task. [30] developed and presented an annotation guideline including different definitions and a flow chart to let the experts distinguish among tweets including ADEs from those that do not. However, they did not provide the measure used for The inter-rater agreement. [7] developed an annotation platform to guide the two experts in medical terminologies to annotate the ADEs relationships. However, the authors did not provide the inter-rater agreement. They just mentioned that In case of disagreements, the annotators discussed to achieve a consensus. If a lot of disagreements occurred, the annotators were asked to learn the guidelines and revise their annotations.

While annotation guidelines remain largely absent in most studies, a few recent works have started to address this gap by proposing more coherent and robust annotation protocols tailored to their specific tasks. Sahoo et al. [108] created the multimodal MMADE dataset combining text and images to enhance adverse drug event (ADE) detection. Although their work focuses on vision-language models and dataset development, they mention using a short annotation guideline for labelling text-image pairs, but the details are limited. [112] annotated VAERS reports specifically for Guillain-Barré Syndrome and highlighted how refining their annotation guideline improved inter-annotator agreement from 69% to 86%. They emphasised lessons learned during the iterative development of these guidelines [112]. Martínez et al. [88] mined Spanish-language Twitter posts for vaccine-related ADEs and used custom annotation guidelines tailored to the informal nature of tweets. The guidelines addressed slang, ambiguity, and annotation agreement, supporting classification tasks using transformer models like RoBERTuito [88]. Dai et al. [24] introduced the MultiADE benchmark spanning social media, clinical notes, and publications. Their CADECv2 dataset was annotated using a comprehensive guideline designed to ensure consistency across domains and to support robust ADEs extraction across contexts [24]. Finally, Dirkson et al. [32] extracted coping strategies from social media posts about ADEs, guided by annotation guidelines that focused on interpreting nuanced, context-rich content. Annotators were trained to handle challenges such as sarcasm and implicit expressions of coping.

Three other research studies also relied on existing annotation guidelines, though not their ones [6, 57, 86]. This used the annotation guidelines from shared tasks (such as the 2009 i2b2 task^22^) and studies presenting supporting documents (such as the Arizona disease Corpus (AZDC) [73] annotation guidelines) to complete their annotation process. To sum up, we can observe that the majority of the studies including an annotation guideline briefly describe it within the paper. It is an open issue to address where the proposed guidelines could be helpful and useful for other research studies within the field.

Preparing a suitable guideline for annotation is as challenging as it is important for developing a standard and uniform dataset. And, this is not only applicable to adverse event detection or medical datasets. It is related to any NLP tasks. For example [90] highlighted the importance of having an annotation guideline for the sentiment analysis task. These authors also highlighted the major challenges related to sentiment annotation, including named entities, modifiers, questions and modalities.^23^. More complex than sentiment analysis, emotion detection (i.e. happiness, sadness, fear, surprise, anger) also requires a robust and coherent annotation guideline. For this purpose, [56] demonstrated how the implementation of complete and comprehensive guidelines for multi-label emotion annotation led to substantially (30%) higher agreement scores among human annotators. These authors highlighted some challenges regarding emotion annotation, including the fine-grained contexts, grammar sensitivity and the annotator’s perspective . The annotation is also an important step for extracting semantic roles. In this context, [15] presented the annotation guideline used for annotating PropBank.^24^ However, the annotation process is fastidious and time-consuming. Hence, the latest research studies are investigating how to achieve a high-quality annotation from non-experts without extensive training. Ref. [46]. In this context, the authors developed a crowdsourcing-friendly coreference annotation methodology (ezCoref) dedicated to coreference annotation. This platform includes an intuitive, open-sourced annotation tool supported by a short, crowd-oriented interactive tutorial. This platform was used to re-annotate 240 passages from different coreference datasets using Amazon Mechanical Turk (AMT). The authors concluded that a high-quality annotation (>90% of the corpora) was achieved from non-expert annotators.

### Word Embedding Models Used

**Fig. 4:**
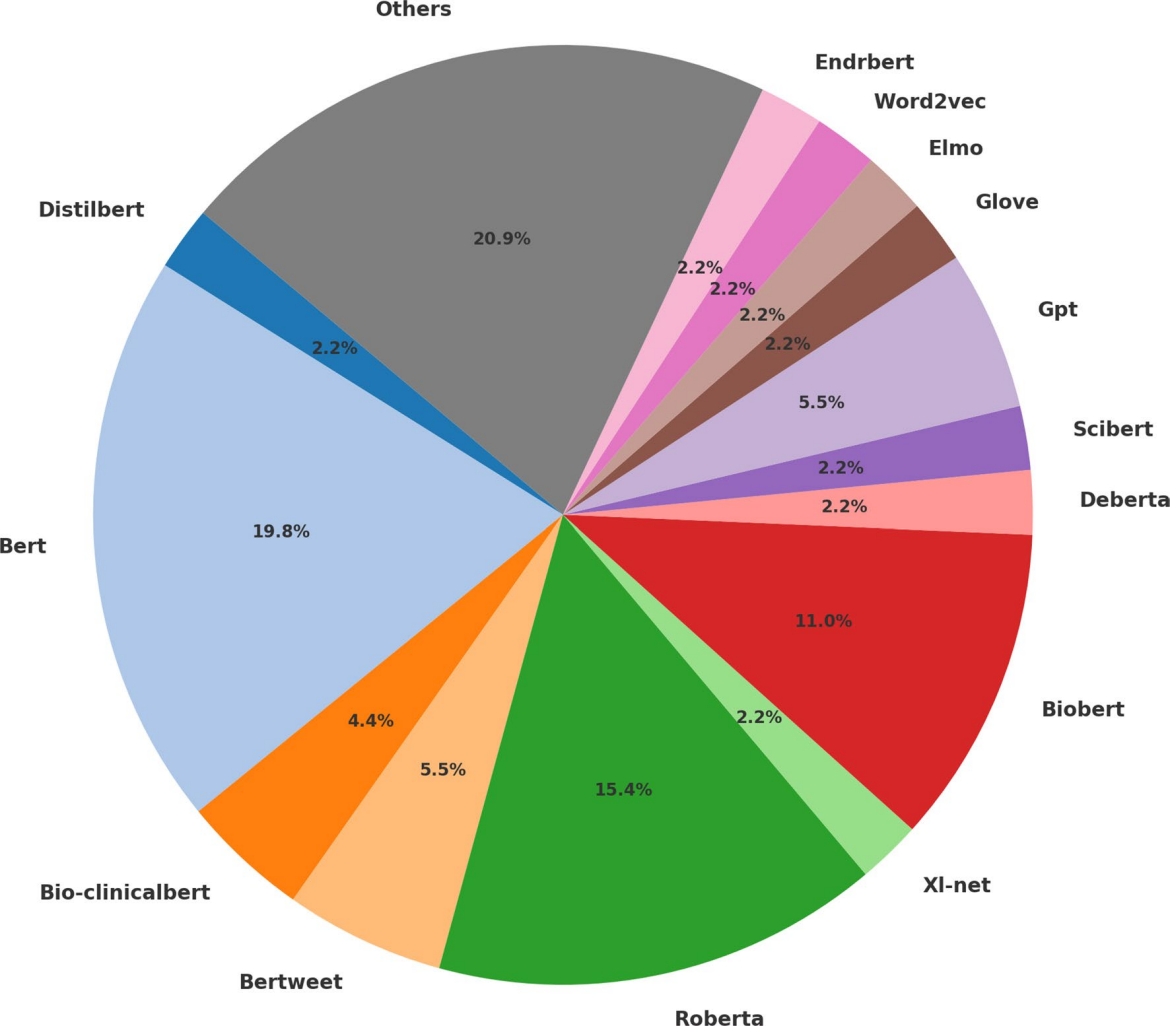
Proportion of the embedding models

In total, 33 (illustrated in Fig. 4) embedding models were used. These models were referenced 91 times within the studied papers. BERT and RoBERTa were the most used models with BERT used in 18/91 (19.8%) studies and RoBERTa in 14/91 (15.4%) studies. BERT is a bidirectional transformer pre-trained using a combination of masked language modeling objective and next sentence prediction on a large corpus comprising the Toronto Book Corpus and Wikipedia. BERT has been popular for different NLP tasks because, unlike recent language representation models, BERT is designed to pre-train deep bidirectional representations from the unlabeled text by joint conditioning on both the left and right context in all layers. As a result, the pre-trained BERT model can be fine-tuned with just one additional output layer to create state-of-the-art models for a wide range of tasks, such as question answering and language inference, without substantial task-specific architecture modifications.^25^ RoBERTa builds on BERT and modifies key hyperparameters, removing the next-sentence pretraining objective and training with much larger mini-batches and learning rates.^26^

In general, we observed that the use of transformers is predominant whereas other approaches mainly involve the use of BERT trained on tweets, clinical text or PubMed. However, we also observed that some studies were still relying on first-generation embedding models such as word2vec (3/76; 3.95%), (2/76; 2.63%), Glove (2/91; 1.1%), FastText (1/91; 5.26%) and Glove (2/91; 2.2%).

### Machine Learning Algorithms, Tools and Libraries

The majority of studies required the use of NLP techniques and machine learning algorithms to perform more advanced tasks such as ADEs detection [28, 33, 58, 80, 119, 122] classification [16, 23, 29, 37, 64, 80, 105] or normalisation [59, 104]. The use of ML allows for solving contextual issues and for automatically finding relations between drugs and side effects. However, their performance highly depends on the approach adopted and on the choice of the right model in the right way as well as on the size of the training data. This is why many studies have been conducted to increase results and improve model performance. Some of them focused on reducing the noise produced [87] where feature reduction was used to improve an SVM model.

**Fig. 5:**
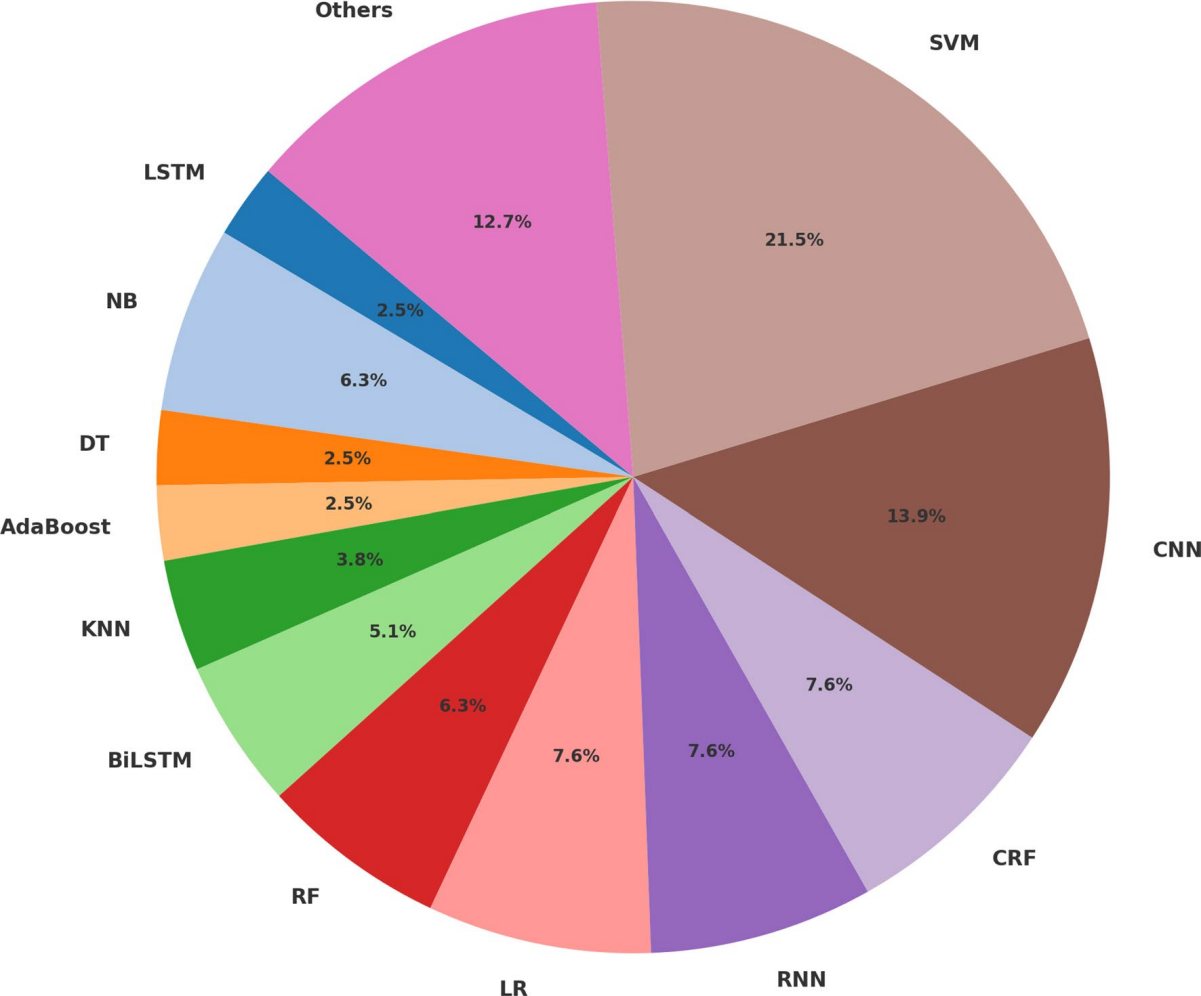
Proportion of the ML algorithms

A considerable number of machine learning algorithms, libraries and tools have been used in the studied papers (24 in total, illustrated in Fig. 5). The authors used different varieties of algorithms and tools, which can be grouped into traditional machine learning algorithms (i.e. SVM, RF [50], DT [91], NB [89], etc.) and deep learning algorithms (i.e. CNN [95], CNN-LSTM, CNN-BiLSTM, etc.). We observed that SVM is the top-used ML algorithm (24/79; 30.4%)). It is followed by CNN (11/79; 13.92%). Some authors relied on existing tools for named entity recognition such as CoreNLP,^27^ OpenNLP^28^ and Spacy.^29^

The majority of corpora used for the detection of ADEs are unbalanced where the number of documents including events is significantly lower than the number of documents without them. To deal with this situation, some authors used under-sampling (such as RUS, RUSB and VUE) or over-sampling (such as SMOTE and WESMOTE) algorithms to balance the dataset before using ML algorithms for training models.

Other approaches based on the use of AI with deterministic approaches were proposed to mitigate the challenges faced in ADE detection [55]. It is not uncommon to rely on sentiment analysis techniques for improving the performance of the classification and the detection models [54, 119]. However, as the performance of a model mainly depends on its training set, different studies highlighted the fact that increasing the number of annotated data or relying on an additional training dataset improves the results returned by a model and its efficiency [72, 87].

Finally, training these models in a time-efficient manner is also challenging; some models need to be trained for weeks. Some useful modern technologies allow applying heavy computing on cloud platforms and reduce the time needed to train our models such as APACHE SPARK [54].

### Drugs Referenced by the Studies

Table 7 lists the number of referenced drugs for each task. Our first observation is related to the limited number of referenced drugs on papers proposing approaches for detecting ADEs. This is mainly because the majority of studies are not specific. Only a few studies target specific drugs whereas the others are designed for different drugs mentioned in the comments. Different recent studies (8/100; 8%) proposed to highlight the adverse vaccine events of Influenza vaccine based on patients’ testimony on social media [10, 20, 36, 48, 58, 75, 78, 103]. We observed that the number of these studies is more frequent than the studies that have been done on Influenza vaccines (only one study). This observation is relevant not only to the Influenza vaccine but to all other drugs listed in Table 7 which were mentioned only once in the reviewed papers. We observed that almost all of the studies are focused on developing NLP techniques to improve patient-centred care.

**Table 7:** The referenced drugs

The referenced drugs	Classification	Detection	Normalisation	Resource construction	Pipeline	Analysis	Total per drug
Flu shot	1	0	0	0	0	0	1
COVID19 vaccine	11	6	0	0	3	2	22
Influenza vaccines	1	0	0	0	0	0	1
Dietary Supplements	0	1	0	0	0	0	1
Acne medication Isotretinoine	0	1	0	0	0	0	1
Diclofenac	0	0	0	1	0	0	1
Lipitor	0	0	0	1	0	0	1
Methylphenidate	0	0	0	0	1	0	1
Statin medication	0	0	0	0	0	1	1
Complementary and Alternatively Medcicine with AutoImmune Hepatitis (CAM-AIH)	0	0	0	0	0	1	1
Antidepressants	0	0	0	0	0	1	1
Acétate de cyprotérone	0	1	0	0	0	0	1
Fluoxétine	0	1	0	0	0	0	1
Méthadone	0	1	0	0	0	0	1
Sofosbuvir	0	1	0	0	0	0	1
Codeine	0	1	0	0	0	0	1
Hydroxyzine	0	1	0	0	0	0	1
Nicorandil	0	1	0	0	0	0	1
Midodrine	0	1	0	0	0	0	1
Galantamine	0	1	0	0	0	0	1
Crizotinib	0	1	0	0	0	0	1
Valproate de sodium	0	1	0	0	0	0	1
Fingolimod	0	1	0	0	0	0	1
Aripiprazole	0	1	0	0	0	0	1
Amoxicillin	2	0	0	0	0	0	2
Ibuprofen	1	2	0	1	0	0	4
Cisplatin	0	1	0	1	0	0	2
Doxorubicin	0	1	0	1	0	0	2
Tamoxifen	0	1	0	1	0	0	2
Zoloft	1	1	0	0	0	0	2
Lexapro	0	1	0	0	0	0	1
Ostarine	0	0	0	0	0	1	1
Ligandrol	0	0	0	0	0	1	1
Testolone	0	0	0	0	0	1	1
Ozempic	0	1	0	0	0	1	2
Wegovy	0	1	0	0	0	1	2
Saxenda	0	1	0	0	0	1	2
Trulicity	0	1	0	0	0	1	2
Sinopharm	1	1	0	0	0	0	2
Metformin	1	0	0	1	0	0	2
Hydrochlorothiazide	1	0	0	0	0	0	1
Aspirin	1	1	0	1	0	0	3
Warfarin	1	1	0	0	0	0	2
Semaglutide	0	1	0	1	0	0	2
Adderall	1	1	0	0	0	0	2
Tylenol	1	1	0	0	0	0	2
Dexamethasone	1	0	0	0	0	0	1
Remdesivir	1	0	0	0	0	0	1
Azithromycin	1	0	0	0	0	0	1
Hydroxychloroquine	0	0	0	0	0	1	1
Tramadol	0	1	0	0	0	0	1
Oxycodone	0	1	0	0	0	0	1
Paracetamol	1	0	0	0	0	0	1
Mounjaro	0	0	0	0	0	1	1
salicylic acid	0	0	0	0	0	1	1
retinoids	0	0	0	0	0	1	1
Sertraline	1	0	0	0	0	0	1
Total	29	40	0	9	4	16	**98**

### Emerging Trends and Challenges

*Comparative performance landscape (across the 100 studies).*

- **Binary classification (tweet/post level):** Classical ML (SVM/LR/NB) typically ACC/F1 $\approx$ 0.75$-$0.86 [37, 54, 125]. CNN/biLSTM models often F1 $\approx$ 0.74$-$0.83 [114, 115]. Transformer baselines (BERT/BERTweet/RoBERTa) commonly F1 $\approx$ 0.80$-$0.92 depending on corpus size/balance and augmentation [2, 26, 48, 63, 100].
- **Span extraction (NER):** BERT-family and RoBERTa variants with CRF/attention achieve F1 $\approx$ 0.78$-$0.86 with external validation on ADE corpora [33, 67, 127].
- **Normalisation (to MedDRA/UMLS):** Neural transition-based methods on tweets remain low (F1 $\sim$0.22) [59]; sentence-transformer biomedical linkers push to F1 $\sim$0.50$-$0.65 across CADEC/PsyTAR/TwiMed [104]. Zero/few-shot LLMs currently trail these linkers on fine-grained codes [140].
- **Imbalance handling:** Synthetic oversampling and cost-sensitive training (e.g., WESMOTE, class weighting, targeted augmentation) consistently improve minority recall in ADE-positive classes [2, 23, 100].

Building on the synthesis above, we highlight seven cross-cutting trends that clarify *how* current methods work in practice, *where* they succeed, and *why* important gaps persist. Where possible, we relate each trend to concrete results reported in the reviewed papers to provide a comparative view of model effectiveness.

*T1. From lexicons and linear models to transformers–and what each still does best.* Early systems relied on lexicons (e.g., UMLS/CHV/MedDRA) and rule-based pipelines, which remain valuable for *coverage*, auditability, and hypothesis generation (e.g., discovery of candidate ADRs and drug-event co-occurrences) [96, 131]. Classical ML (SVM, LR, NB) with BoW/TF-IDF features performs competitively on clean, balanced corpora and remains strong baselines for binary ADE classification: e.g., SVM and ensembles achieved ACC/F1 in the 0.77$-$0.86 range in large Twitter cohorts [37, 54, 125]. CNN/biLSTM models typically improve span sensitivity and context use, yielding F1 around 0.74$-$0.83 for tweet-level detection/extraction [114, 115, 121]. Transformer fine-tuning (BERT, RoBERTa, BERTweet, BioClinicalBERT) is now the default for both *classification* (F1 $\approx$ 0.80$-$0.92 on SMM4H-style tasks) and *extraction* (NER F1 $\approx$ 0.78$-$0.86 with external validation) [2, 26, 33, 48, 63, 67, 100]. In short: lexicons maximise recall and interpretability; linear models are robust and fast; deep sequence models capture local context; transformers provide the strongest general performance.

*T2. Joint and end-to-end pipelines help–yet error propagation remains a bottleneck.* Joint architectures that share representations across *classification*, *extraction*, and *normalisation* mitigate cascading errors and exploit task synergies [31, 36, 109]. Despite this, normalisation remains the weakest link: tweet-to-MedDRA mapping is still challenging given slang, implicit mentions, and ambiguity. Reported normalisation scores remain modest (e.g., F1 $\approx$ 0.18$-$0.50 in shared-task settings) [31, 36], with recent sentence-transformer approaches pushing the state of the art into the 0.50$-$0.65 range across CADEC/PsyTAR/TwiMed [104]. These results underline a persistent gap between strong mention detection and reliable concept linking in noisy, consumer language.

*T3. Multimodality and knowledge integration improve* *precision* *and support verification.* A growing thread augments text with images and/or biomedical knowledge to reduce false positives and enrich interpretation. Vision-language models and text+image datasets (e.g., MMADE) improve detection and summarisation quality on posts that visually substantiate adverse experiences [108]. On the knowledge side, linking social media signals with FAERS/openFDA, SIDER, DRUGO, or UMLS (and using NER toolkits such as SciSpaCy) strengthens validation and supports cluster/co-occurrence analyses [9, 25, 72, 128]. Recent work also builds *knowledge graphs* of ADEs from Reddit with LLMs to structure free-text reports [34]. Overall, hybrid text+knowledge pipelines tend to trade a small recall loss for improved precision and actionability.

*T4. Multilingual and cross-domain generalisation is still fragile.* Performance commonly drops when moving *across* languages or domains (forums $\leftrightarrow$ Twitter $\leftrightarrow$ clinical notes). Studies in Arabic and Spanish show that careful pretraining and regional models (e.g., RoBERTuito/BETO) recover much of the loss but not all [4, 88]. Cross-lingual BERT-family models help on Russian/English [109], and Chinese lexicon+sequence approaches demonstrate feasibility [137]. The MultiADE benchmark quantifies domain-shift penalties across social media, clinical notes, and publications, with the best in-domain systems still losing ground out-of-domain [24]. Template-based robustness probes likewise expose sensitivity to lexical/grammatical variations [85]. Practical systems therefore benefit from domain-adaptive pretraining, lightweight continual learning, and judicious use of distant supervision.

*T5. Temporal and longitudinal modelling for early warning remains underused.* Most ADE systems treat posts as independent and identically distributed (i.i.d) datapoints. However, credible adverse experiences unfold over time (symptom onset $\rightarrow$ persistence $\rightarrow$ resolution or switching medication). Graph/sequence models (e.g., GAR, credibility-aware Bayesian models) begin to capture temporal/co-mention structure and authenticity [51, 113]. Severity estimation from large embedding spaces (SAEDR) provides an orthogonal, population-level trend measure [72]. Integration with surveillance data (VAERS), topic dynamics, and graph mining uncovers both known and previously undocumented vaccine effects [136]. Large-scale sentiment/NER timelines over pandemic-era tweets further illustrate how longitudinal signals can be exploited [76]. Despite encouraging results, end-to-end, timeline-aware ADE detectors are still rare.

*T6. Credibility, quality, bias, and ethics: necessary checks before use in pharmacovigilance.* Veracity varies widely; explicit protocols and inter-annotator agreement help, but systematic credibility modelling is needed for deployment. Data-veracity annotation and credibility-aware models reduce spurious flags [51, 84]. Cross-source triangulation indicates that social media signals can agree with official sources for common drug-event pairs, while also surfacing novel signals [9, 43, 142]. Platform demographics (e.g., Twitter’s age skew) pose representativeness concerns [65]. Quality audits of user-generated video (e.g., TikTok) report low understandability/actionability overall, underscoring misinformation risks [116]. Ethical analyses caution that public acceptability, privacy, and consent must be addressed explicitly [42]. These findings argue for built-in bias audits, privacy-preserving processing, and human-in-the-loop review for high-impact signals.

*T7. LLMs and quantum-inspired models: promise with caveats.* LLM-powered pipelines appear in two roles: (i) as *classifiers/extractors* and (ii) as *retrievers/synthesisers* over authoritative documents. As standalone ADE classifiers on tweets, fine-tuned ChatGPT-style models have achieved competitive F1 (e.g., $\sim$0.75), but remain behind top RoBERTa baselines (F1 $\sim$0.80) on SMM4H datasets [26]. For normalisation, zero/few-shot LLMs underperform specialised linkers (e.g., GPT-4 F1 $\sim$0.31 vs. BioLORD/SapBERT-style approaches $\sim$0.50$-$0.65), reflecting the difficulty of precise MedDRA mapping [104, 140]. In contrast, *retrieval-augmented* LLMs over FDA drug labelling can deliver highly accurate safety profiling (F1 $\ge$0.91) in document-grounded settings [130]. Quantum and hybrid quantum-classical architectures report strong headline metrics on curated review datasets (F1/ACC $\sim$0.90$-$0.97), but replication on noisy, open-domain social media remains limited [28, 29]. Overall: LLMs excel when grounded by high-quality retrieval; for tweet-level supervised tasks, specialised transformers still set the pace.

*Practical gaps and opportunities.* First, improved *concept linking* and *explainability* are pivotal for regulatory use: severity scoring [72], relation extraction [127], interpretable graph/fuzzy methods [25], and attention/saliency inspection are complementary ways to make outputs reviewable. Second, *temporal* and *user-timeline* modelling should become standard to prioritise persistent and escalating signals [51, 113, 136]. Third, *multilingual/domain-adaptive* pretraining and benchmarks such as MultiADE are essential to close generalisation gaps [4, 24, 85]. Finally, for real-world deployment, the most promising pattern is *retrieval-augmented, knowledge-linked* transformers/LLMs with calibrated confidence, human-in-the-loop adjudication, and routine bias/quality audits [42, 43, 130, 142].

## Practical Applications of Findings in Pharmacovigilance and Regulatory Practices

Social media can help health authorities find possible side effects of drugs earlier than traditional methods. Unlike official reporting systems, social media gives fast and constant access to real-time patient experiences. Studies such as [25, 131] show that NLP tools can identify early warning signs from user posts. Organizations like the FDA or EMA can include social media monitoring tools in their systems to track new or growing safety concerns, allowing faster investigations or safety alerts.

Many patients do not report their side effects through official systems, either because they are unaware of them or because reporting is too complicated. According to [14, 127], people are more likely to talk about their experiences on social media. These platforms offer a simpler and more informal way to learn about how people react to medications. Regulators can use social media to collect more complete information, especially from people who usually don’t report side effects through formal systems.

Sometimes, new side effects appear only after a drug is widely used by the public. Studies like [72, 130] show that social media can provide useful information after a drug is on the market. This information can be linked to medical codes (like MedDRA) to help experts review and update safety labels. egulators can use social media findings to decide if a drug’s label should be changed – for example, to include new warnings or dosage advice.

Social media can help public health officials understand how drug side effects spread over time and in different places. [112] shows how this data can help track vaccine side effects, for example. The insights can guide decisions about where to focus health campaigns or how to respond to public concerns. Health agencies can create better communication plans and health policies based on what they learn from public discussions on platforms like Twitter and Reddit. Social media data, when combined with hospital systems, can also support better clinical decisions. For example, [33] show that combining social media findings with clinical data can help detect side effects more accurately. Hospitals and healthcare software can include ADE alerts in their systems to warn doctors about possible risks when prescribing medications.

Pharmaceutical companies can benefit from monitoring social media to understand how patients feel about their products. They can also detect off-label use, new side effects, or reasons why patients stop taking certain drugs. Studies like [9] show that some side effects found on social media had not been reported in official sources. Drug companies can improve their post-marketing research and make better decisions about product development and risk communication. Many researchers now use common datasets like SMM4H and CADEC to train and compare their models. As [31, 140] point out, this makes it easier to compare results and improve systems. Health regulators can set up testing rules using these datasets to check how reliable ADE detection tools are before approving them for official use.

## Discussion and Future Direction

This paper presents a literature review highlighting the most recent approaches, tools, models and datasets that were presented for detecting ADEs from social media using NLP. For extracting our studies, we queried *Google Scholar*. We were interested in all the papers related to the extraction of ADEs from social media. Hence, we collected the papers including our search query (ADEs/ADR/adverse drug event/adverse drug reaction/side effect, etc. and social media) within the title.

In addition to the title search, we relied only on *Google Scholar* for extracting our papers. This could also lead to excluding some relevant papers published in other libraries such as *Scopus*, *ACM* or *IEEE* and that we did not include. We also did not rely on any tools such as *Covidence*^30^ for preparing this literature review. We only used Word and Excel for screening and data extraction. The de-duplication was then done manually. As the number of papers collected initially was not voluminous (130 papers initially sought), it was manageable to remove duplicated papers manually. However, as we carried out a literature review and not a systematic review, no protocol was proposed. The papers were initially selected by one reviewer. The full-text screening was done by one reviewer and the data extraction was carried out by different reviewers. The data extraction pipeline was defined in Excel.

In order to improve this work, we are currently working on a systematic review, where we defined a protocol and where we are querying six distinct databases (Embase, Medline, Web Of Science, ACM Guide to Computing Literature, IEEE Digital Library and Scopus). For this systematic review, we are using *Covidence* and de-duplication was done automatically. Also, we have three reviewers for each step where two of them are screening and extracting the data in parallel and the third one is resolving conflicts. Finally, for this ongoing systematic review, we target broader datasets, where we are not only interested in the detection of ADEs in social media. We are also interested in the extraction of ADEs from free text in general (including data from social media, discharge summaries, General practitioner notes, etc.).

Our main observation from this paper is that despite growing advances in detecting Adverse Drug Events (ADEs) from social media using NLP, several research gaps remain. Below are key future directions, each supported by prior studies cited in this review.

### Expanding Beyond English and Twitter

The majority of the studies reviewed in this work focused on Twitter, with English as the primary language of analysis. This narrow focus limits the generalizability of ADE detection systems. As noted in [14], and further evidenced in multilingual works like [4], there is a clear need to expand to other platforms such as Reddit, Facebook, and regional health forums, and to support additional languages like Arabic, French, or Chinese. Multilingual approaches, as demonstrated by [85] and [88], show promising results with models such as RoBERTuito and XLM-RoBERTa. Future systems should explore transfer learning and cross-lingual embeddings to detect ADEs across language boundaries.

### Improving Annotation Practices and Guidelines

Inconsistent or absent annotation guidelines remain a critical issue. Only a minority of the reviewed works report using formal guidelines during annotation (e.g., [88, 112]. This undermines reproducibility and the ability to compare models across datasets. Annotation ambiguity–especially for implicit ADEs–has been highlighted in [31, 59]. Future research should prioritize the creation of standardized, domain-informed annotation protocols and measure inter-annotator agreement systematically. The integration of expert-driven labeling and public annotation tools such as BRAT or Knowtator [6] is also recommended.

### Multimodal and Multisource Integration

While textual analysis dominates the field, emerging studies demonstrate the value of integrating multiple data types. For instance, [108] leverage text-image pairs using vision-language models like LSTM+ResNet50 and GIT, achieving state-of-the-art scores on multimodal benchmarks. Similarly, [9] combine Reddit, Twitter, and PubMed data, enriching ADE detection with structured biomedical information. Such integrative approaches should be extended to include structured sources like FAERS [25], or knowledge graphs built from LLMs [34] . These hybrid pipelines could help bridge the semantic gap between user-generated content and clinical evidence.

### Explainability and Trustworthiness of Models

As models become more complex–especially with transformer-based architectures like RoBERTa, BioBERT, and GPT$-$3.5 [79, 119] –their interpretability becomes increasingly important. [127] emphasize the challenge of validating ADE relations from social media without insight into model decision-making. Future research must adopt explainable AI (XAI) techniques, such as attention heatmaps or SHAP values, particularly for high-stakes applications. This aligns with ongoing concerns in health AI more broadly, where explainability is a prerequisite for regulatory acceptance and clinical use.

### Temporal and Longitudinal Analysis

Most current ADE systems process social media posts in isolation. However, symptoms often develop over time, requiring temporal modeling to capture causality or chronic side effects. While longitudinal analysis remains underexplored, [51, 113] introduce early models that utilise sequential data and attention mechanisms, hinting at the potential for timeline-aware ADE surveillance. Building user-level timelines across multiple posts–especially using graph embeddings or time-aware transformers–could improve real-time pharmacovigilance and early warning systems.

### Domain Adaptation and Few-/Zero-Shot Learning

The fast pace of drug development and evolving public health crises (e.g., COVID-19) demand models that generalise to unseen scenarios. [29, 33] have demonstrated strong performance using transformer models and hybrid architectures for generalisation. Meanwhile, [83] employ question-answering frameworks that could be adapted for zero-shot inference. Future work should focus on developing robust few-shot learning pipelines and domain-adaptive pretraining (e.g., continual learning with BioClinicalBERT) to ensure adaptability to emerging ADEs.

### Ethical Considerations and Bias Mitigation

Demographic skew and lack of diversity in social media data remain pressing issues. As noted in [65], younger users dominate Twitter, leading to unbalanced population representation. Moreover, works like [48] show that keyword-based filtering can reinforce biases. Ethical ADE systems must incorporate bias audits, fairness metrics, and privacy-preserving techniques (e.g., de-identification, differential privacy). Additionally, models should be stress-tested across different demographics and linguistic styles to ensure equitable performance.

### Real-World Deployment and Collaboration with Regulatory Bodies

While many models show high F1-scores in controlled benchmarks ( [29, 102]) , few are integrated into real-world pharmacovigilance pipelines. Collaboration with regulatory bodies like the FDA, MHRA, and WHO is essential to validate these systems against real-world data and reporting workflows. Studies such as [130] show the feasibility of deploying large language models with retrieval mechanisms for real-time ADE profiling. Future work should emphasize system integration, user interface design, and clinical validation to ensure practical uptake.

## Comparison with Existing Reviews

Several recent reviews discuss adverse event detection and pharmacovigilance from complementary vantage points, but differ from our scope and granularity. Golder et al. [41] present a scoping review of *social media analysis for adverse event detection*, emphasising utility, feasibility, and overarching challenges (e.g., data quality, ethics, integration with signal detection). In contrast, our review delivers a *task-structured, model-centric synthesis* across 100 studies (2017–2025), spanning binary/multilabel classification, sequence labelling for extraction, ontology-driven normalisation (e.g., MedDRA/UMLS/SNOMED CT), end-to-end pipelines, resource construction, and downstream analytical studies. We provide side-by-side comparisons of classical ML, CNN/RNN/CRF, and transformer/LLM variants (BERT, BioBERT, RoBERTa, BERTweet), including strategies for class imbalance and augmentation, external validation on widely used benchmarks (e.g., SMM4H, CADEC, TwiMed), and a fine-grained error typology (implicit ADEs, figurative language, sarcasm).

Golder et al. [44] scope *NLP and ML for ADE detection in EHR/EMR text*, focusing on clinical-document pipelines (section detection, negation, concept extraction) and governance issues specific to clinical narratives. Our review is complementary: we concentrate on *open-domain user-generated content* (Twitter/X, Reddit, forums) and its distinct methodological pressures (noisy consumer vocabulary, conversational context, platform conventions), while extending the analysis to *normalisation from consumer phrasing to clinical ontologies* and cross-platform robustness.

Broader pharmacovigilance surveys such as Salas et al. [110] synthesise the *use of AI in pharmacovigilance* across case processing, signal detection, and workflow automation. They provide a wide operational lens, whereas our contribution offers *deep coverage of NLP methods* specifically for ADE detection from social media, including multimodal (text+image) settings, multilingual coverage, emerging LLM- and quantum-inspired models, temporal modelling, and practical pathways for *regulatory integration*. Finally, the perspective on patient and public involvement by van Hunsel et al. [123] underlines engagement, ethics, and representativeness—concerns we operationalise by quantifying annotation practice and agreement, documenting dataset biases, and proposing concrete reporting and linkage practices (e.g., consistent use of MedDRA coding) to translate social-media signals into pharmacovigilance workflows.

*Distinct contributions of this review.* (i) A task-structured synthesis (classification, extraction, normalisation, pipelines, resources, analyses) with *comparative model effectiveness* across families and benchmarks; (ii) the most detailed coverage to date of *normalisation* from noisy consumer mentions to standard ontologies and joint extraction+linking pipelines; (iii) explicit quantification of *annotation practices* (guideline availability, IAA) and class-imbalance handling; (iv) integration of *multilingual and multimodal* advances alongside LLM/quantum-inspired methods and temporal modelling; and (v) *practice-facing guidance* mapping findings to pharmacovigilance and regulatory use.

## Conclusion

Detecting Adverse Drug Events (ADEs) from social media using Natural Language Processing (NLP) has evolved into a dynamic and impactful research area. This literature review analyzed 100 peer-reviewed studies published between 2017 and 2025, highlighting the range of methods, datasets, and challenges involved in ADE classification, extraction, normalization, and analysis.

Our findings confirm that Twitter is still the most widely used social media platform in this domain, with the vast majority of studies focused on English-language data. Transformer-based models, particularly BERT and its variants such as RoBERTa, BioBERT, and BERTweet, dominate current approaches due to their strong performance across tasks. Pre-processing techniques like URL removal, tokenization, and lowercasing are almost universally applied, and data imbalance remains a key issue, particularly when detecting rare or implicit ADE mentions. Despite technical progress, significant challenges persist. Annotation of ADEs, especially those involving subtle or implicit drug-event relationships, is still inconsistent and under-documented. Many studies rely heavily on shared datasets such as SMM4H, but these are often limited in scope, language, and real-world diversity. Multilingual support, cross-platform data integration, and temporal modeling of patient health narratives remain underexplored but promising areas.

Recent trends show increasing interest in multimodal approaches, explainable AI, and real-world validation. However, deployment in regulatory settings and integration into clinical workflows is still limited. For ADE detection systems to have meaningful real-world impact, future research must address these challenges while promoting collaboration with regulatory bodies, healthcare providers, and multilingual user communities.

## Data Availability

N/A

## Abbreviations

ACC: Accuracy
AE: Adverse event
AEFI: Adverse event following immunization
ADE: Adverse drug event
ADR: Adverse drug reaction
AI: Artificial intelligence
AKNN: Adaptive *k*-nearest neighbors
ALBERT: A lite BERT
AUC: Area under the ROC curve
BERT: Bidirectional encoder representations from transformers
BERTweet: BERT model pretrained on Twitter
BioBERT: BERT pretrained on biomedical text
Bio_ClinicalBERT: BERT pretrained on clinical text
BioELECTRA: ELECTRA pretrained on biomedical text
BLIP: Bootstrapping language-image pretraining (vision-language)
BoW: Bag-of-words
BTM: Biterm topic model
CADEC: Corpus of adverse drug event annotations
CADECv2: Updated CADEC corpus
CAM: Complementary and alternative medicine
CHV: Consumer health vocabulary
CNN: Convolutional neural network
CRF: Conditional random field
cTAKES: Clinical text analysis and knowledge extraction system
DeBERTa: Decoding-enhanced BERT with disentangled attention
DeepWalk: Random-walk-based graph embedding
DL: Deep learning
DT: Decision tree
EHR: Electronic health record
EL: Entity Linking
ELMo: Embeddings from language models
ERNIE: Enhanced representation through knowledge integration
FAERS: FDA adverse event reporting system
FDA: U.S. Food and Drug Administration
F1: F1-score (harmonic mean of precision and recall)
FastText: Subword-aware word embeddings
GAT: Graph attention network
GATE: General architecture for text engineering
GIT: Vision-language transformer for image-text tasks
GLP-1 RA: Glucagon-like peptide-1 receptor agonist
GloVe: Global vectors for word representation
GRU: Gated recurrent unit
kNN: *k*-nearest neighbors
LDA: Latent Dirichlet allocation
LLM: Large language model
LN(S): Layer normalization (and variants)
LR: Logistic regression
LSTM: Long short-term memory
MCEM: Monte Carlo expectation-maximization (signal detection)
MedDRA: Medical dictionary for regulatory activities
MedLEE: Medical language extraction and encoding system
ML: Machine learning
NB: Naive Bayes
NER: Named entity recognition
Node2Vec: Biased random-walk graph embedding
NLP: Natural language processing
P: Precision
P@10: Precision at rank 10
PCA: Principal component analysis
PSB2016: 2016 Patient Safety Benchmark dataset (Twitter)
PubMedBERT: BERT pretrained solely on PubMed
QA: Question answering
R: Recall
RAG: Retrieval-augmented generation
RF: Random forest
RE: Relation extraction
RNN: Recurrent neural network
RoBERTa: Robustly optimized BERT pretraining approach
RoBERTuito: Spanish Twitter-pretrained RoBERTa
RUS: Random under-sampling
SAGE: Sparse additive generative model
SBERT: Sentence-BERT (sentence embeddings)
SciBERT: BERT pretrained on scientific text
SDNE: Structural deep network embedding
SMM4H: Social media mining for health (shared task)
SMOTE: Synthetic minority over-sampling technique
SRS: Spontaneous reporting system
SVM: Support vector machine
STS: Semantic textual similarity
TF-IDF: Term frequency-inverse document frequency
TwiMed: Twitter + PubMed ADE corpus
TwitterADR: Twitter adverse drug reaction dataset
UMLS: Unified medical language system
VAERS: Vaccine adverse event reporting system
VADER: Valence Aware Dictionary and sEntiment Reasoner
VQC: Variational quantum circuit
VUE: Under-sampling variant used in imbalanced learning pipelines
WESMOTE: Word-embedding-based SMOTE
word2vec: Neural word embeddings (CBOW/Skip-gram)
XLNet: Generalized autoregressive pretraining for language understanding

## Footnotes

1 https://www.nlm.nih.gov/research/umls/index.html.

2 https://www.snomed.org/.

3 https://www.meddra.org/.

4 https://yellowcard.mhra.gov.uk.

5 https://scholar.google.com.

6 https://developer.twitter.com/en/docs/twitter-api.

7 https://www.medhelp.org/.

8 https://www.fda.gov/drugs/drug-approvals-and-databases/fda-adverse-event-reporting-system-faers.

9 https://www.nlm.nih.gov/research/umls/sourcereleasedocs/current/CHV/sourcerepresentation.html.

10 https://gate.ac.uk/family/training.html.

11 https://www.ncbi.nlm.nih.gov/pmc/articles/PMC7165158/

12 https://data.gov.au/dataset/ds-dap-csiro%3A10948/details?q=

13 http://bio-nlp.org/index.php/projects/39-nlp-challenges.

14 http://bio-nlp.org/index.php/projects/39-nlp-challenges.

15 https://data.mendeley.com/datasets/rxwfb3tysd/2.

16 https://huggingface.co/datasets/bookcorpus.

17 https://huggingface.co/datasets/cc_news.

18 https://huggingface.co/datasets/openwebtext.

19 Central nervous system stimulant drug used to treat attention deficit hyperactivity disorder and, to a lesser extent, narcolepsy.

20 Vaccine Adverse Event Reporting System (VAERS).

21 https://www.fda.gov/.

22 https://www.i2b2.org/NLP/DataSets/Main.php.

23 Sentiment Analysis (SA) is a Natural Language Processing (NLP) and an Information Extraction (IE) task that primarily aims to obtain the writer’s feelings expressed in positive or negative terms by analysing a large number of documents. [90].

24 a corpus in which the arguments of each predicate are annotated with their semantic roles about the predicate [97]

25 https://huggingface.co/docs/transformers/model_doc/bert.

26 https://huggingface.co/docs/transformers/model_doc/roberta.

27 https://stanfordnlp.github.io/CoreNLP/.

28 https://opennlp.apache.org/.

29 https://spacy.io/.

30 https://www.covidence.org/.

## Declarations

### Research Involving Human and/or Animals

N/A
